# Cancer and Environmental Xenobiotics: Mechanisms, Controversies, and Innovations

**DOI:** 10.3390/jox16010002

**Published:** 2025-12-19

**Authors:** Alice N. Mafe, Dietrich Büsselberg

**Affiliations:** 1 Department of Biological Sciences, Faculty of Sciences, Taraba State University, Main Campus, Jalingo 660101, Taraba State, Nigeria; mafealice1991@gmail.com; 2 Department of Physiology and Biophysics, Faculty of Medicine, Qatar Campus, Weill Cornell Medicine-Qatar, Education City, Qatar Foundation, Doha Metropolitan Area, Doha P.O. Box 22104, Qatar

**Keywords:** epigenetics, tumor microenvironment, public health, persistent organic pollutants, molecular toxicology, oxidative stress

## Abstract

Although cancer biology has advanced considerably, the impact of environmental toxins on carcinogenesis remains underrecognized and scattered across disciplines. Evidence increasingly shows that chronic exposure to a broad range of toxins—including persistent organic pollutants, heavy metals, pesticides, phthalates, microplastics, and fine particulate matter (PM2.5), which significantly contributes to cancer initiation, progression, and treatment resistance. This review synthesizes mechanistic, molecular, and epidemiological findings from 2015 to 2025, identified through systematic searches of PubMed, Scopus, Web of Science, and MeSH. Key pathways include oxidative stress-mediated DNA damage, epigenetic reprogramming (DNA methylation, histone modifications, miRNA dysregulation), hormone receptor modulation, chronic inflammation, immune evasion, and tumor microenvironment remodeling. Case studies of benzene, arsenic, aflatoxins, pesticides, and microplastics detail exposure routes, molecular targets, and associated cancers, highlighting significant public health risks. Ongoing debates persist regarding safe exposure thresholds, latency periods, and the effects of mixed toxin exposures. The review also highlights recent innovations in environmental oncology, including AI-based predictive models, CRISPR screens for susceptibility genes, organoid/3D models, green chemistry interventions, and real-time exposure monitoring, which provide mechanistic insight and inform early detection and personalized prevention strategies. Additionally, regional data gaps, particularly in low- and middle-income countries, indicate the need for stronger interdisciplinary collaboration. By integrating molecular mechanisms, epidemiology, and technological advances, this review offers a comprehensive framework for understanding toxin-induced carcinogenesis and guiding future research, public health policy, and preventive strategies.

## 1. Introduction

Cancer continues to rank among the foremost causes of mortality worldwide [1]. Although genetic factors are well established in driving tumor development, increasing attention is being directed toward the role of environmental exposures in triggering and accelerating cancer progression [2]. Pollutants, dietary toxins, and other chemical agents are now recognized as critical contributors that extend beyond genetic predisposition in shaping cancer risk [3].

Given this recognition, research into cancer biology and environmental toxicology has progressed mainly in parallel rather than in synergy. This separation has limited the ability to understand how toxins influence the molecular pathology of cancer fully. Integrating these two areas of study is crucial for understanding how environmental agents affect genetic and cellular mechanisms, potentially influencing susceptibility, progression, and treatment response. However, several knowledge gaps remain unresolved. These include uncertainties in dose–response dynamics, the cumulative effects of long-term low-dose exposures, and the complexities of latency periods [4], and the impact of multiple toxins acting in combination [5]. In particular, the scarcity of region-specific toxicological and epidemiological data in low- and middle-income countries (LMICs) has created major blind spots in risk assessment and policy formulation, thereby limiting global understanding of environmental carcinogenesis [6,7]. In addition, epidemiological data often blur the line between correlation and causation, sparking debate and leaving key questions unanswered [8].

This review focuses on specific categories of environmental toxins most strongly implicated in human cancers, including industrial chemicals, pesticides, and persistent organic pollutants. These groups include substances recognized by the International Agency for Research on Cancer (IARC) [9] and the World Health Organization (WHO) as established or probable carcinogens and thereby marked their global health significance [10]. This review adopts a transdisciplinary perspective, integrating toxicological evidence with oncological mechanisms to provide a more comprehensive understanding of cancer risks associated with environmental exposures. Such an approach mirrors real-world conditions, where individuals are rarely exposed to a single toxin in isolation but rather to complex mixtures over extended periods.

The discussion centers on the classes of environmental toxins most strongly linked with cancer, such as endocrine-disrupting chemicals, heavy metals, airborne contaminants, and food-derived toxins. Their mechanisms of action, as well as both supportive and conflicting findings, and recent advances in detection and intervention strategies, are critically examined. The primary aim of this work is to evaluate the multifaceted interplay between environmental toxins and cancer development. By emphasizing the public health implications of toxin exposure, particularly in vulnerable populations and poorly regulated regions, this review denotes the urgent need for integrated prevention frameworks. By synthesizing evidence across disciplines, this review offers fresh insights into mechanistic pathways and highlights opportunities for translational research. Ultimately, it emphasizes the potential of integrating toxicological knowledge into oncology to advance cancer prevention, early detection, and therapeutic innovation.

## 2. Methodology

**Figure 1:**
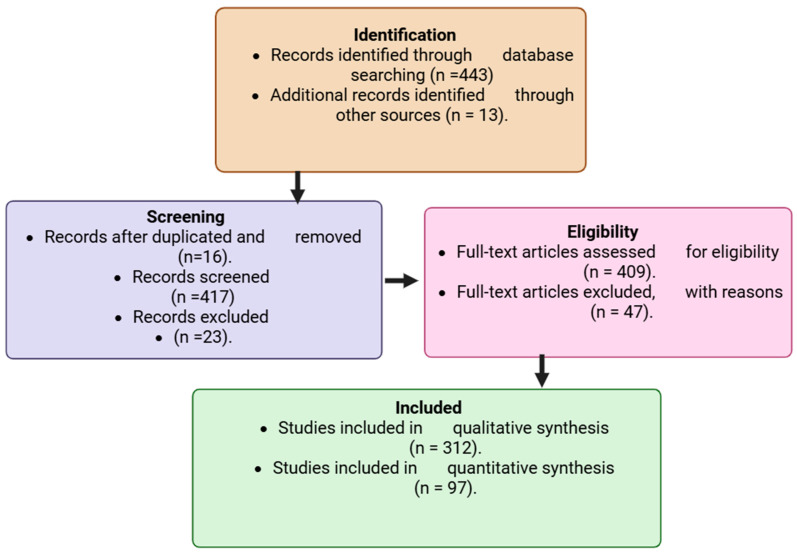
Flowchart showing the study selection process for inclusion in the review. Created in BioRender. Busselberg, D. (2025) https://BioRender.com/omql52t. acceesed on 12 November 2025.

A comprehensive literature search was conducted across PubMed (https://pubmed.ncbi.nlm.nih.gov/ accessed 2 August 2025), Scopus (https://www.scopus.com) accesed 7 August 2025, and Web of Science (https://www.webofscience.com accessed 11 August 2025) databases, covering the period 2015–2025. These databases were selected for their extensive indexing of peer-reviewed biomedical, toxicological, and oncological studies. To refine and broaden the search strategy, Medical Subject Headings (MeSH) terms were used in combination with carefully selected keywords linking environmental toxins to cancer. The search strategy incorporated Boolean operators (“AND,” “OR,” and “NOT”) to combine relevant keywords and MeSH terms. The main search terms included: “environmental carcinogens,” “industrial chemicals,” “pesticides,” “persistent organic pollutants,” “heavy metals,” “airborne contaminants,” “endocrine disruptors,” “food-derived toxins,” “tumorigenesis,” and “cancer risk.” These combinations were adapted to each database’s indexing system to ensure comprehensive retrieval. Inclusion criteria were limited to peer-reviewed original research articles, experimental studies, systematic reviews, and meta-analyses that directly examined associations between environmental toxins and cancer outcomes. Only studies published in English between 2015 and 2025 were considered to ensure contemporary relevance. Exclusion criteria encompassed commentaries, editorials, non-peer-reviewed sources, and papers without mechanistic or epidemiological data linking toxins to cancer, to maintain scientific rigor and reliability. All retrieved articles were imported into reference management software to remove duplicates. Screening was conducted in two stages: (1) title and abstract screening, followed by (2) full-text evaluation. Two independent reviewers screened and cross-verified eligible studies to minimize bias and ensure consistency. A flow diagram summarized the selection process, including the number of records retrieved per search term, those screened, those excluded, and those finally included for complete analysis. Eligible studies were systematically categorized by toxin class, cancer type, underlying biological mechanism, and overall strength of evidence. This structured organization facilitated cross-comparison of environmental exposures and their oncogenic potential. Each study underwent critical evaluation focusing on methodological rigor, reproducibility, novelty, and its contribution to the mechanistic understanding of toxin-driven carcinogenesis. This rigorous and transparent approach ensured that both well-established findings and emerging insights were captured, forming a scientifically credible foundation for the integrative discussion presented in this review. The flow chart diagram, as shown, indicates the identification, screening, exclusion, and those included in the study. The study selection process for inclusion in the review is illustrated in Figure 1.

## 3. Key Mechanisms of Toxin-Induced Carcinogenesis

Environmental toxins promote carcinogenesis through several interrelated biological processes [11]. These include oxidative damage, epigenetic remodeling, endocrine disruption, chronic inflammation, changes in the tumor microenvironment, and the emergence of distinct mutational patterns [12]. Each mechanism contributes uniquely to cancer initiation and progression, while often acting in concert to drive malignant transformation [13].

### 3.1. Oxidative Stress and DNA Damage

A primary mechanism by which toxins exert carcinogenic effects is through oxidative stress [14]. Agents such as heavy metals, persistent organic pollutants, and airborne particulates trigger the excessive production of reactive oxygen species (ROS) and reactive nitrogen species (RNS) [15]. When antioxidant defenses are overwhelmed, oxidative damage accumulates in cellular components, especially DNA [16]. Thus, DNA damage emerges as a key downstream consequence of persistent oxidative stress, linking environmental toxin exposure directly to genomic instability and mutagenesis [17,18,19]. This results in strand breaks, base modifications, and cross-linking, all of which undermine genomic stability.

Sustained oxidative stress not only promotes mutations in tumor suppressors and oncogenes but also activates signaling cascades such as NF-κB and MAPK that enhance cell proliferation and resistance to apoptosis [20]. Mitochondrial dysfunction caused by toxins further amplifies ROS generation, establishing a feedback loop that accelerates DNA damage and carcinogenic transformation [21].

#### Genotoxicity and DNA Repair Mechanisms

**Figure 2:**
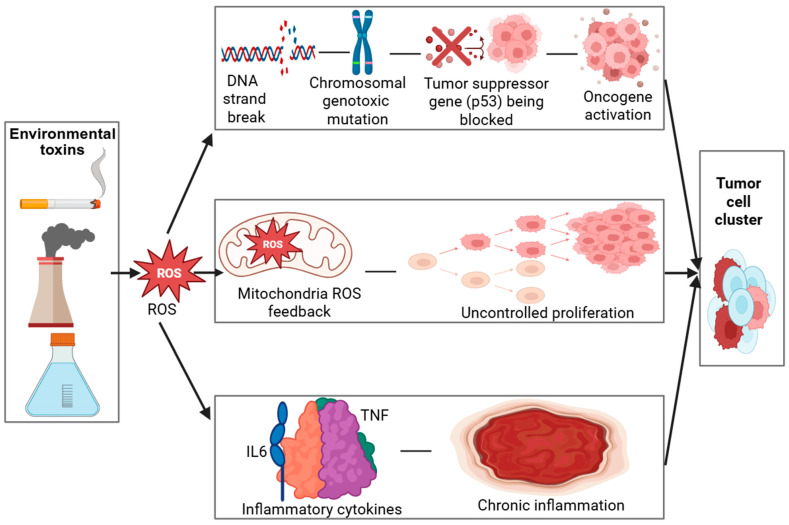
Created in BioRender. Busselberg, D. (2025) https://BioRender.com/inf6tqo. accesed on 12 December 2025. Legend: DNA—Deoxyribonucleic acid, ROS—Reactive oxygen species, p53—Tumor Protein p53 (Guardian of the Genome), IL6—Interleukin 6, TNF—Tumor necrosis factor. Mechanisms of Oxidative Stress–Mediated Carcinogenesis.

In addition to inducing oxidative lesions, many environmental toxins possess direct genotoxic potential, primarily through the formation of DNA adducts and reactive intermediates that disrupt the helical structure and compromise replication fidelity [22,23]. These genotoxic insults can result in point mutations, chromosomal aberrations, and microsatellite instability, which are recognized as molecular hallmarks of early-stage carcinogenesis [24,25]. Under normal physiological conditions, DNA repair pathways such as base excision repair (BER), nucleotide excision repair (NER), and mismatch repair (MMR) function to preserve genomic integrity [26]. However, chronic or repeated toxin exposure can suppress or overwhelm these repair mechanisms, leading to incomplete or error-prone repair [27,28]. The progressive accumulation of unrepaired or misrepaired DNA lesions thereby increases the mutational burden within cells, ultimately promoting genomic instability and malignant transformation. As shown in Table 1, the mechanisms of oxidative stress–mediated carcinogenesis involve multiple cellular pathways. The role of oxidative stress in carcinogenesis is further detailed through a visual model (Figure 2), which demonstrates how ROS-induced DNA damage activates oncogenic pathways while impairing tumor suppressors.

Environmental toxins (industrial emissions, cigarette smoke, pollutants) trigger oxidative stress, leading to excessive ROS (Reactive Oxygen Species) production. ROS induce DNA (Deoxyribonucleic Acid) strand breaks and mutations, impair p53 (Tumor Protein p53—the “Guardian of the Genome”) function, and activate oncogenes, resulting in uncontrolled cell proliferation. Mitochondrial ROS amplify oxidative stress through a positive feedback loop. Elevated TNF (Tumor Necrosis Factor) and IL-6 (Interleukin 6) drive chronic inflammation, further promoting tumor development.

### 3.2. Epigenetic Modifications (DNA Methylation, Histone Modifications, miRNA Dysregulation)

Beyond direct genetic mutations, toxins also induce heritable changes in gene expression through epigenetic reprogramming [37]. DNA methylation patterns are particularly affected, exposures to arsenic, cadmium, and certain pesticides have been linked to global hypomethylation as well as gene-specific hypermethylation that silences tumor suppressor pathways [38]. Mechanistically, these toxins alter the activity of DNA methyltransferases (DNMTs), leading to aberrant methylation of cytosine residues within CpG islands, which in turn suppresses transcription of genes critical for cell cycle regulation and apoptosis [39,40,41].

Histone alterations add another layer of regulation. By modifying acetylation and methylation states, toxins remodel chromatin architecture, often in ways that favor unchecked cell growth [42]. Environmental toxins such as heavy metals and polycyclic aromatic hydrocarbons can disrupt histone acetyltransferase (HAT) and histone deacetylase (HDAC) activity, causing changes in chromatin compaction that either repress or activate oncogenic gene expression [43,44,45].

Additionally, non-coding RNAs, especially microRNAs (miRNAs), are disrupted by pollutants and endocrine-disrupting chemicals [46]. Dysregulated miRNAs promote angiogenesis, epithelial–mesenchymal transition, and metastatic potential [47]. Toxins may interfere with miRNA transcription or processing enzymes such as DROSHA and DICER, leading to abnormal miRNA expression profiles that influence post-transcriptional regulation of tumor suppressor and oncogene networks [48].

Importantly, since epigenetic modifications are reversible, they hold promise as biomarkers of exposure and targets for preventive or therapeutic intervention. Epigenetic reprogramming caused by environmental toxins demonstrates how pollutants can modify DNA methylation, histone architecture, and miRNA expression, ultimately influencing tumor behavior (Table 2).

### 3.3. Hormone Receptor Activation and Endocrine Disruption

Endocrine-disrupting chemicals (EDCs) such as bisphenol A, phthalates, and organochlorine pesticides interfere with natural hormone signaling [70]. By binding to estrogen, androgen, or thyroid receptors, these compounds mimic or block hormonal activity, leading to abnormal signaling in hormone-sensitive organs like the breast, prostate, and thyroid [71]. Mechanistically, EDCs can act as agonists or antagonists at estrogen receptors (ERα and ERβ), androgen receptors (ARs), or thyroid hormone receptors (TRs), thereby altering receptor conformation, recruitment of coactivators or corepressors, and downstream transcriptional activity [72,73].

For instance, xenoestrogens enhance estrogen receptor activity in breast tissue, stimulating proliferation even in the absence of endogenous hormones [74]. In prostate cancer, EDCs disrupt androgen receptor pathways and contribute to resistance against hormonal therapies [75]. Animal studies have shown that prenatal or early-life exposure to bisphenol A increases mammary gland density and susceptibility to tumor formation [76,77,78]. In contrast, human epidemiological studies link chronic phthalate exposure to elevated risks of breast and prostate cancers [79,80,81]. Some toxins also alter hormone synthesis, metabolism, or clearance, compounding their carcinogenic effects through long-term endocrine imbalance [82].

In addition to synthetic chemicals, certain heavy metals such as cadmium, arsenic, and lead also exhibit endocrine-disrupting activity [83]. Cadmium, often described as a “metalloestrogen,” can bind to estrogen receptors and activate estrogenic signaling pathways in breast and uterine cells, thereby promoting uncontrolled proliferation [84]. Arsenic has been shown to interfere with glucocorticoid and thyroid hormone signaling, while lead exposure has been associated with altered reproductive hormone levels and impaired feedback mechanisms in the hypothalamic–pituitary–gonadal axis [85]. Experimental evidence from rodent models demonstrates that chronic cadmium exposure induces mammary gland hyperplasia and estrogen-responsive gene activation [86,87], while population-based studies reveal associations between urinary cadmium levels and increased breast cancer incidence [88,89]. These metal-induced disruptions further highlight the multifaceted nature of endocrine imbalance in carcinogenesis.

### 3.4. Inflammation and Immune Evasion

Chronic inflammation represents another key route of toxin-driven carcinogenesis [90]. Substances such as asbestos, silica, and air pollutants activate innate immune responses that sustain low-grade, persistent inflammation [91]. Pro-inflammatory cytokines like IL-6, TNF-α, and IL-1β create a milieu that promotes proliferation, angiogenesis, and survival of transformed cells [92]. Beyond inorganic particulates and metals, several organic pollutants, such as polycyclic aromatic hydrocarbons (PAHs), dioxins, and persistent organic pollutants, have also been implicated in maintaining chronic inflammatory signaling, further broadening the spectrum of inflammation-inducing agents [93,94].

Over time, inflammatory conditions enhance genomic instability through continuous ROS and RNS production [95]. In parallel, toxins compromise immune surveillance by inducing T-cell dysfunction, expanding regulatory T-cell populations, and impairing antigen presentation [96]. This establishes a mechanistic continuum in which chronic inflammation fosters an immunosuppressive tumor microenvironment. Persistent cytokine signaling and oxidative stress not only promote DNA damage but also drive immune exhaustion, enabling malignant cells to evade cytotoxic T-cell recognition [97,98,99]. Consequently, the interplay between inflammation and immune evasion becomes self-reinforcing as toxins sustain inflammatory stimuli that facilitate oncogenic mutations [100,101], while immune escape mechanisms allow emerging tumor cells to persist and expand unchecked [102,103]. This dual effect includes perpetuating inflammation while weakening anti-tumor immunity, allowing premalignant cells to evade detection and progress toward malignancy [104].

### 3.5. Tumor Microenvironment Alterations

Environmental toxins also reshape the tumor microenvironment (TME), a critical regulator of cancer dynamics [105]. Exposures can stimulate extracellular matrix remodeling, fibroblast activation, and abnormal angiogenesis via increased VEGF signaling [106]. Arsenic and cadmium, for example, are known to enhance blood vessel formation and stromal support, thereby facilitating tumor growth and spread [107].

Beyond heavy metals, other environmental contaminants such as fine particulate matter (PM2.5), pesticides, and industrial chemicals can also disrupt the structural integrity of the ECM and promote aberrant tissue remodeling [108,109]. These agents stimulate matrix metalloproteinases (MMPs) and fibronectin deposition, weakening cell–cell adhesion and promoting tumor invasion [110].

Toxin exposure also reprograms immune cells within the TME. Macrophages often shift toward the tumor-promoting M2 phenotype, while myeloid-derived suppressor cells accumulate, dampening anti-tumor immunity [111]. Such exposures may also influence cancer-associated fibroblasts (CAFs), enhancing their secretion of growth factors and cytokines that support tumor–stromal crosstalk, angiogenesis, and metastatic spread [112,113].

Hypoxia, worsened by toxin-induced vascular dysfunction, further activates HIF-1α pathways that drive invasion and therapeutic resistance [114]. Together, these changes transform the microenvironment into a supportive niche for cancer progression.

Beyond structural and immune alterations, metal exposures further destabilize tumor microenvironments by disrupting calcium homeostasis and redox balance [115]. Arsenic trioxide, for example, has been shown to interfere with calcium signaling in both tumor and non-tumor cells, inducing cell-type-specific cytotoxicity at environmentally and clinically relevant concentrations [116]. Such disruptions affect intracellular communication, apoptosis regulation, and stress responses within the TME.

Additionally, oxidative imbalance triggered by heavy metals like cadmium and lead intensifies reactive oxygen species accumulation, which not only damages DNA but also reinforces a chronic inflammatory state [117]. Similarly, chronic exposure to PM2.5 and pesticide residues has been linked to persistent oxidative stress and endothelial dysfunction, promoting sustained angiogenesis and stromal remodeling within the TME [118,119]. This oxidative stress creates a feedback loop that sustains angiogenesis, fibroblast activation, and immune evasion, ultimately establishing a microenvironment more permissive to cancer progression [120].

### 3.6. Toxin-Induced Mutational Signatures

Perhaps the most direct evidence linking toxins to cancer is found in mutational signatures, which are distinct genomic patterns left by specific exposures. Aflatoxin B1 [121], for instance, induces a characteristic G → T transversion in codon 249 of TP53, a hallmark of hepatocellular carcinoma [122]. Tobacco smoke produces well-documented C → A transversions, while arsenic exposure correlates with widespread chromosomal instability [123]. It is essential to distinguish between “toxins,” which are biologically produced agents such as aflatoxins from fungi, and “toxicants,” which refer to human-made or synthetic chemicals such as benzene, polycyclic aromatic hydrocarbons, and industrial solvents [124]. This distinction provides conceptual clarity in understanding the diverse sources of mutagenic exposures.

**Figure 3:**
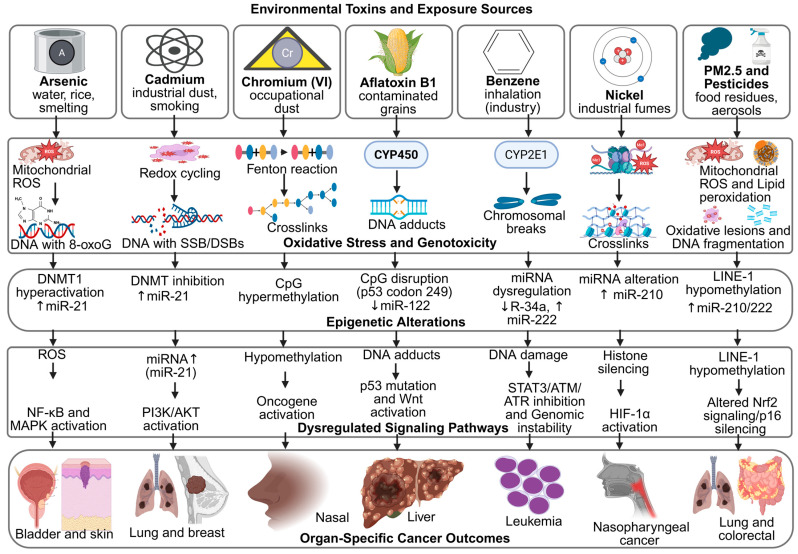
Mechanistic Links Between Environmental Toxins, Oxidative Stress, Epigenetic Remodeling, and Cancer Outcomes. Created in BioRender. Busselberg, D. (2025) https://BioRender.com/ta3gsrk. accessed on 12 November 2025. Legend: AFB1—Aflatoxin B1, ATM/ATR—Ataxia Telangiectasia Mutated/ATM and Rad3-related, BPA—Bisphenol A, BRCA1—Breast Cancer 1 Gene, CpG—Cytosine–Phosphate–Guanine site, CYP450/CYP2E1—Cytochrome P450 Enzymes, DNMT—DNA Methyltransferase, DSB—Double-Strand Break, ERα—Estrogen Receptor Alpha, H3K4me2/H3K9ac/H3K27me3—Histone Modification Marks, HIF-1α—Hypoxia-Inducible Factor 1-alpha, LINE-1—Long Interspersed Nuclear Element-1, MAPK/ERK—Mitogen-Activated Protein Kinase/Extracellular Signal-Regulated Kinase, miRNA—MicroRNA, MLH1/CDH1/p16—Tumor-Suppressor Genes, NF-κB—Nuclear Factor kappa-light-chain-enhancer of activated B cells, Nrf2—Nuclear Factor Erythroid 2–Related Factor 2, OPs—Organophosphate Pesticides, PAHs—Polycyclic Aromatic Hydrocarbons, PI3K/AKT—Phosphatidylinositol-3-Kinase/Protein Kinase B, PM2.5—Particulate Matter ≤ 2.5 µm, ROS—Reactive Oxygen Species, SSB—Single-Strand Break, STAT3—Signal Transducer and Activator of Transcription 3, Wnt/β-catenin—Canonical Wnt Signaling Pathway.

These signatures serve as molecular fingerprints, confirming the causal relationship between exposure and carcinogenesis [125]. For example, benzene exposure is associated with characteristic chromosomal aberrations and point mutations in hematopoietic malignancies, while aflatoxin B1 produces a distinct TP53 mutation spectrum unique to liver cancer [126]. Similarly, polycyclic aromatic hydrocarbons from combustion sources leave identifiable DNA adduct patterns that can be traced to specific exposure histories [127,128]. Advances in next-generation sequencing have expanded the catalog of such patterns, enabling more precise attribution of cancers to environmental causes [129]. Moreover, identifying mutational signatures may inform therapeutic decisions, as tumors with specific exposure-driven mutations can exhibit distinct treatment responses [130]. These interconnected mechanisms are further summarized in Figure 3, which highlights the convergence of oxidative stress and epigenetic dysregulation in toxin-induced carcinogenesis.

## 4. Case Studies: Linking Specific Toxins to Cancer

**Figure 4:**
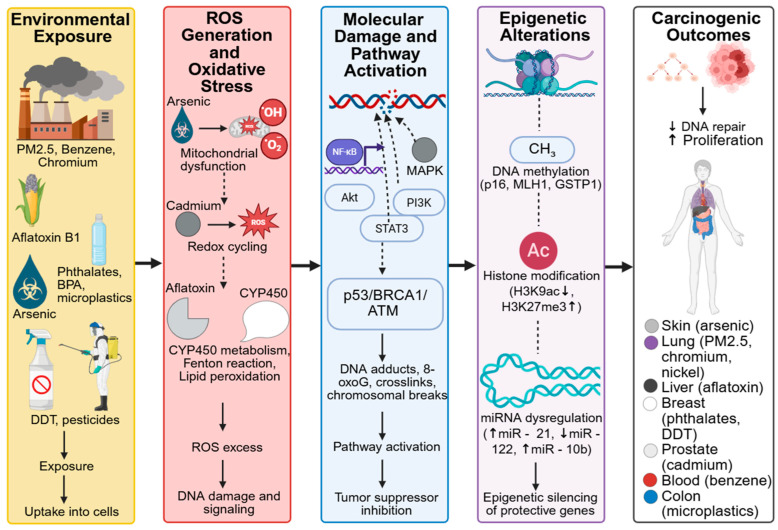
Mechanistic Links Between Environmental Toxins, Oxidative Stress, Epigenetic Reprogramming, and Carcinogenesis. Created in BioRender. Busselberg, D. (2025) https://BioRender.com/r8cnd0f. accesed on 12 December 2025. Legend: Cellular-level targets include mitochondria, DNA, and histones—where toxins induce oxidative stress, DNA methylation changes, and chromatin remodeling. Tissue-level effects involve liver damage, chronic inflammation, fibrosis, and remodeling of the tumor microenvironment, collectively promoting malignant transformation and cancer progression.

To illustrate the real-world impact of environmental toxins on cancer development, it is essential to examine specific examples where strong mechanistic and epidemiological evidence converge. Case studies offer concrete insights into how individual toxins contribute to particular cancer types, highlighting both molecular pathways and their public health implications. The following subsections examine well-documented toxins, including benzene, aflatoxins, arsenic, phthalates, airborne PM2.5, and microplastics, and explore their modes of action in relation to cancer risk and progression. Case studies across global populations highlight how different exposure routes, such as ingestion, inhalation, and dermal contact, mediate toxin-specific cancer outcomes (Table 3). Figure 4 depicts the mechanistic links between environmental toxins, oxidative stress, epigenetic reprogramming, and carcinogenesis.

### 4.1. Benzene and Hematologic Malignancies

Benzene is a widely used industrial chemical and is a well-established human carcinogen strongly linked to hematologic cancers, particularly acute myeloid leukemia [154]. The primary routes of exposure include inhalation of contaminated air in industrial or traffic-dense environments and dermal absorption through contact with petroleum products or solvents [155,156]. Chronic benzene exposure disrupts hematopoietic stem cell function, induces chromosomal aberrations, and promotes oxidative stress, collectively driving malignant transformation [157]. Mechanistically, benzene metabolites, such as benzoquinones, generate reactive oxygen species, induce DNA strand breaks, and interfere with topoisomerase II, leading to genomic instability and bone marrow toxicity [158,159]. Epidemiological data from occupational and environmental settings consistently confirm this association, underscoring the urgent need for stringent exposure controls [160]. Beyond acute myeloid leukemia, benzene exposure has also been associated with aplastic anemia and other hematologic malignancies, including non-Hodgkin lymphoma and myelodysplastic syndromes [161].

### 4.2. Aflatoxins and Liver Cancer

Aflatoxins are toxic metabolites produced by *Aspergillus* species [162] and are among the most potent naturally occurring carcinogens [163]. The primary route of exposure is ingestion through contaminated grains, nuts, and other food products, particularly under warm and humid storage conditions [164,165]. Their impact is most evident in hepatocellular carcinoma, where they form DNA adducts and induce mutations in the TP53 gene [166]. Specifically, aflatoxin B1 generates a characteristic G → T transversion at codon 249 of the TP53 gene, serving as a molecular hallmark of aflatoxin-induced liver cancer [167]. Mechanistically, the toxin exerts its carcinogenic effect through the formation of reactive epoxides that bind to DNA, oxidative stress generation, and interference with DNA repair pathways [168,169]. The risk is further amplified when exposure co-occurs with hepatitis B infection, creating a synergistic effect [170]. Despite regulatory monitoring of food supplies, aflatoxins continue to pose a significant health risk in regions with inadequate food safety practices. In addition to hepatocellular carcinoma, chronic aflatoxin exposure has been associated with increased risk of gallbladder and gastrointestinal cancers in some populations [171].

### 4.3. Arsenic and Skin/Bladder Cancer

Chronic exposure to inorganic arsenic, primarily through contaminated groundwater, is linked to skin and bladder cancers [172]. The predominant exposure route is ingestion via contaminated drinking water, although inhalation and dermal absorption can also occur in occupational or mining settings [173]. Arsenic interferes with DNA repair, induces oxidative stress, and alters epigenetic regulation, which contribute to its carcinogenic potential [174]. Mechanistically, arsenic promotes oxidative stress through mitochondrial dysfunction and ROS overproduction, leading to DNA strand breaks and mutagenic lesions [175]. It also impairs base and nucleotide excision repair pathways and causes widespread epigenetic dysregulation, such as aberrant DNA methylation and histone modification, that silence tumor suppressor genes [176]. Epidemiological studies in affected regions such as Bangladesh, India, and parts of Latin America highlight the strong association between long-term arsenic exposure and cancer incidence [177], making it a pressing global health concern [178]. Beyond skin and bladder cancers, chronic arsenic exposure has also been implicated in lung, kidney, and liver cancers due to its systemic distribution and cumulative toxicity [179].

### 4.4. Phthalates and Breast/Prostate Cancer

Phthalates are commonly used as plasticizers in consumer products and are recognized as endocrine-disrupting chemicals [180]. Major exposure routes include ingestion of contaminated food and water, dermal absorption from cosmetics and personal care products, and inhalation of indoor dust containing phthalate residues [181,182]. They mimic or interfere with natural hormones, thereby influencing estrogen and androgen signaling pathways [183]. Mechanistically, phthalates act through hormonal disruption by binding to estrogen receptors (ERs) and androgen receptors (ARs), modulating downstream gene expression involved in cell proliferation and apoptosis. They also induce oxidative stress and may interfere with epigenetic regulation, amplifying their carcinogenic potential [184]. Evidence suggests that chronic phthalate exposure may promote breast and prostate carcinogenesis by enhancing hormone receptor activation and altering gene expression [81]. Although debates remain regarding exposure thresholds, their widespread use raises significant public health concerns. Epidemiological and experimental studies have increasingly linked high urinary phthalate metabolite levels with elevated risks of hormone-dependent cancers, reinforcing their role as environmental contributors to carcinogenesis [185].

### 4.5. Airborne PM2.5 and Lung Cancer

Particulate matter (PM2.5), a key component of air pollution, is strongly associated with lung cancer incidence and mortality [186]. The primary exposure route is inhalation, allowing fine particles to penetrate deeply into the respiratory tract and alveolar regions [187,188]. Due to their small size, these particles penetrate deeply into the lungs, causing chronic inflammation, oxidative DNA damage, and activation of oncogenic signaling pathways [189]. Mechanistically, PM2.5 induces oxidative stress, DNA strand breaks, and aberrant DNA methylation in lung epithelial cells, which together promote genomic instability and malignant transformation [190,191]. In addition, PM2.5 exposure activates inflammatory signaling cascades such as NF-κB and STAT3, leading to sustained cytokine production and a tumor-promoting microenvironment [192,193]. Long-term exposure, especially in urban and industrialized environments, has been directly correlated with increased lung cancer risk, as supported by large-scale epidemiological studies [194]. Emerging evidence also links PM2.5 to epigenetic dysregulation and altered gene expression profiles in airway tissues, reinforcing its role as both a mutagenic and epigenetic carcinogen [195].

### 4.6. Microplastics and Colorectal Inflammation-Linked Carcinogenesis

Microplastics, emerging pollutants from plastic degradation, are increasingly implicated in gastrointestinal health risks [196]. Primary exposure occurs through ingestion (via contaminated food and water) and inhalation of airborne microplastic particles [197,198]. Recent evidence suggests that ingestion of microplastics can disrupt gut microbiota, trigger chronic intestinal inflammation [199], and potentially contribute to colorectal carcinogenesis [200]. While mechanistic data are still evolving, the combination of persistent inflammation and genotoxic stress provides a plausible link. Given the global scale of plastic pollution, understanding the long-term health effects of microplastics remains a top research priority. Moreover, emerging studies indicate that microplastics may be associated with multiple cancer types beyond colorectal cancer and can influence additional mechanistic pathways, including oxidative stress, DNA damage, and immune modulation.

### 4.7. Heavy Metals and Cancer

Heavy metals are a diverse group of elements that play a paradoxical role in human health [201]. While essential metals such as zinc, copper, and iron support vital biochemical processes, chronic exposure to toxic metals such as cadmium, arsenic, lead, and chromium has been consistently associated with cancer development [202]. Their capacity to act as both micronutrients and toxicants, depending on concentration and exposure route, makes them particularly challenging from a toxicological perspective [203]. Major exposure routes include ingestion (via contaminated water and food), inhalation of industrial or occupational emissions, and, to a lesser extent, dermal contact through polluted soil or dust [204]. Human exposure occurs through contaminated water, food, industrial emissions, and occupational activities such as mining and smelting [205]. A comprehensive review of their occurrence, uses, and toxic effects underscores their importance as environmental carcinogens [206].

At the mechanistic level, heavy metals disrupt multiple cellular pathways that contribute to carcinogenesis [207]. Their carcinogenicity primarily arises from redox imbalance leading to oxidative stress, inhibition of DNA repair systems, and direct genotoxic effects through DNA adduct formation and chromosomal damage [208]. Cadmium acts as a “metalloestrogen,” binding to estrogen receptors and driving hormone-dependent proliferation [209]. Arsenic impairs DNA repair, induces oxidative stress, and reprograms epigenetic regulation, collectively weakening genomic stability [210]. Lead interferes with calcium signaling and hormone regulation [211], while chromium (VI) undergoes intracellular reduction to reactive intermediates that form DNA crosslinks and strand breaks, initiating mutagenesis [212]. These converging mechanisms, such as oxidative damage, impaired DNA repair, and genotoxicity, facilitate both cancer initiation and progression, contributing to resistance to therapy in exposed populations.

Epidemiological studies further support these mechanistic insights by linking heavy metal exposures to site-specific cancers [213]. Cadmium has been associated with lung and prostate cancer, arsenic exposure through drinking water is strongly tied to skin, bladder, and liver cancers, and chromium (VI) is a well-established occupational carcinogen for lung cancer [214]. Chronic lead exposure, meanwhile, has been connected to increased risks of kidney and brain tumors [215]. Collectively, these associations reinforce the role of both ingestion and inhalation as dominant exposure pathways, with oxidative and genotoxic mechanisms serving as central drivers of malignancy. Such evidence highlights the urgent need for strict regulatory measures to limit both environmental and occupational exposures.

### 4.8. Pesticides and Multiple Cancer Types

Pesticides are among the most widely distributed environmental contaminants, extensively used in agriculture and public health programs to control pests and enhance crop productivity [216,217]. Beyond DDT, other commonly studied pesticides such as organophosphates, carbamates, pyrethroids, and herbicides like glyphosate have also been linked to multiple cancer types. Despite their benefits, mounting evidence indicates that chronic or repeated exposure to certain pesticide classes is associated with an increased risk of multiple cancer types [218]. Human exposure occurs primarily through dermal absorption during handling and application, inhalation of aerosols or contaminated air, and ingestion of pesticide residues on crops and food products [219,220]. These routes contribute cumulatively to the body’s toxic load, particularly among agricultural workers, rural populations, and consumers in regions with high pesticide use.

At the mechanistic level, pesticides promote carcinogenesis through endocrine disruption, genotoxicity, oxidative stress, and epigenetic reprogramming [221,222]. Endocrine-disrupting compounds can mimic or block natural hormones such as estrogen and androgen, altering cellular proliferation and differentiation pathways that facilitate tumor initiation [223]. Genotoxic pesticides induce DNA damage, mutations, and chromosomal instability, while oxidative stress arising from excessive reactive oxygen species can cause lipid peroxidation and DNA strand breaks [224,225]. Epigenetic changes, including aberrant DNA methylation and histone modification, further contribute to altered gene expression and sustained oncogenic signaling [226,227].

Epidemiological evidence supports these mechanistic findings, linking pesticide exposure to leukemia, non-Hodgkin lymphoma, prostate cancer, and breast cancer [217]. Agricultural workers and populations residing near farmlands show higher incidences of these malignancies, reflecting a dose-dependent relationship between long-term exposure and cancer risk [228]. Hematologic cancers, such as leukemia and lymphoma, are particularly associated with organophosphate and carbamate pesticides, whereas endocrine-related cancers like prostate and breast cancers have been linked to persistent organic pollutants and their metabolites [229].

From a public health perspective, the pervasive presence of pesticide residues in food and the environment highlights the urgent need for stricter regulatory enforcement, continuous biomonitoring, and the adoption of safer agricultural practices. Preventive approaches such as integrated pest management (IPM), use of biodegradable and less persistent alternatives, and enhanced occupational safety measures can significantly reduce exposure risks. Continued interdisciplinary research integrating molecular, toxicological, and epidemiological data remains crucial for elucidating the complex biological pathways involved in pesticide-induced carcinogenesis and for guiding evidence-based interventions to safeguard human health. The carcinogenic potential of specific environmental chemicals, along with their IARC classification, is summarized in Table S1, providing a reference for the compounds discussed and in mixed exposure contexts.

From a broader public health perspective, the carcinogenic risks posed by heavy metals call for proactive interventions [230]. Reducing industrial emissions, improving water and food safety, and monitoring occupational exposures remain key strategies [231]. Advances in biomonitoring and molecular epidemiology now make it possible to detect early biological alterations caused by heavy metals, opening opportunities for timely preventive actions [232]. Continued research is crucial for distinguishing the effects of individual metals from those of combined exposures, as most populations are exposed to complex mixtures rather than single agents.

## 5. Critical Evaluation of Existing Research and Controversies

### 5.1. Threshold Effects vs. No-Threshold Models

A central controversy in cancer toxicology is whether carcinogens act according to a threshold effect, where risk is negligible below a specific dose, or a no-threshold model, which argues that even the smallest exposures may contribute to cancer development [233]. This lack of consensus complicates regulatory decisions, as policymakers must choose between precautionary measures and evidence-based dose limits in the face of scientific uncertainty [234].

From an analytical perspective, specific toxicants such as essential trace metals like zinc and copper demonstrate threshold behavior, as physiological homeostasis and detoxification systems can effectively manage low-level exposures [235,236]. Conversely, genotoxic carcinogens such as arsenic, benzene, and ionizing radiation are generally described as non-threshold agents, meaning that even minimal doses may initiate cumulative molecular damage through DNA adduct formation, oxidative stress, or chromosomal instability [237,238].

Distinguishing between causal and correlational evidence remains essential within this framework. Controlled laboratory and mechanistic studies tend to establish causal links by directly showing mutagenic or epigenetic alterations, whereas most epidemiological investigations reveal statistical associations that do not always confirm direct causation [239,240]. Therefore, combining insights from mechanistic toxicology, dose–response modeling, and long-term population studies is necessary to improve the accuracy and reliability of cancer risk assessments [241,242].

### 5.2. Latency Period Challenges

The long latency period between exposure and cancer onset presents another significant difficulty. Many carcinogenic effects may take years or even decades to manifest, which obscures exposure–outcome relationships in epidemiological studies [243]. As a result, risks are frequently underestimated, and preventive measures may be delayed until significant harm has already occurred.

Latency periods differ considerably across toxin categories and exposure intensities, influenced by variations in metabolic detoxification, DNA repair efficiency, and cumulative oxidative stress [244,245]. For example, leukemia linked to chronic benzene exposure may develop within a few years, whereas cancers caused by asbestos or arsenic often take several decades to manifest, even after exposure has ceased [246]. Experimental research using molecular biomarkers and controlled animal models provides stronger causal evidence by demonstrating direct mutagenic or epigenetic alterations from toxin exposure, whereas population-based epidemiological studies typically reveal associative patterns that reflect delayed disease onset [247,248]. Latency-related inconsistencies have been observed in diverse cases, including aflatoxin-induced liver cancer, pesticide-associated non-Hodgkin lymphoma, and heavy metal–linked bladder cancer, highlighting the intricate temporal dynamics that complicate carcinogen risk evaluation [249,250].

### 5.3. Mixed Exposures and Synergistic Effects

Real-world exposures rarely occur in isolation. Individuals are often exposed to multiple toxins simultaneously or sequentially, which can produce synergistic or antagonistic effects that are not captured in controlled laboratory settings [251]. For example, the combined effect of aflatoxin exposure and chronic hepatitis B infection markedly increases liver cancer risk, underscoring the complexity of multifactorial exposures that remain poorly integrated into existing risk models [252].

In cancer toxicology, certain chemicals function as initiators by causing direct DNA damage [253], while others act primarily as promoters by supporting the growth and survival of already altered cells [254]. Classic initiators such as aflatoxin B1 and polycyclic aromatic hydrocarbons induce mutagenic DNA adducts that create genetically vulnerable cells [255]. By contrast, compounds such as dioxins, phorbol esters, and some polychlorinated biphenyls primarily act as tumor promoters, stimulating chronic inflammation, altering key signaling pathways, and disrupting normal cell-cycle control without exerting direct genotoxic effects [256,257]. These mechanistic distinctions become especially important when considering real-world chemical mixtures, because humans are rarely exposed to a single compound in isolation. People encounter combinations of substances that may each occur at low or seemingly safe concentrations in everyday life through food, water, air, and consumer products. Yet when an initiator and a promoter are encountered together, their biological effects can accumulate or even intensify, producing a far stronger carcinogenic response than either could elicit alone. For instance, an initiator that induces early DNA lesions may create pre-mutated cells that become highly susceptible to the proliferative or pro-inflammatory signals generated by a promoter [258]. Consequently, cancer risk cannot be reliably predicted by evaluating individual chemicals separately. Practical hazard assessment must instead consider how distinct mechanisms, such as genotoxicity, oxidative stress, chronic inflammation, endocrine disruption, or epigenetic modulation, interact within complex exposure scenarios. This recognition underscores the need for mixture-based risk assessment frameworks capable of capturing the synergistic or potentiating effects arising from combined exposures, even when single-agent levels appear low.

Multiple investigations have revealed that combinations of environmental contaminants, such as heavy metals, pesticides, and airborne particulates, can produce additive or synergistic interactions that significantly intensify carcinogenic effects [259,260]. For example, co-exposure to arsenic and cadmium has been found to amplify oxidative stress and DNA damage beyond the effects of either compound alone, while simultaneous exposure to phthalates and bisphenol A enhances endocrine disruption and promotes oncogenic signaling cascades [261]. Experimental research using in vivo and in vitro systems provides causal evidence for these synergistic mechanisms by identifying shared molecular pathways and stress responses, whereas large-scale epidemiological studies predominantly yield correlational data linking combined exposures to heightened cancer incidence [262,263]. Collectively, these insights emphasize the urgent need for comprehensive risk assessment models that account for complex chemical mixtures reflective of real-world exposure scenarios.

### 5.4. Inter-Individual Genetic Susceptibility

Genetic diversity further complicates the picture of toxin-induced carcinogenesis. Variations in detoxification enzymes, DNA repair pathways, and immune regulation influence how individuals metabolize and respond to carcinogens [264]. For instance, polymorphisms in cytochrome P450 genes can heighten vulnerability to benzene or aflatoxin-related cancers [265]. Several studies have demonstrated that variants in GSTM1, GSTT1, and NAT2 genes modify the metabolism of polycyclic aromatic hydrocarbons and nitrosamines, thereby altering cancer susceptibility [266,267,268].

Importantly, evidence differentiates between associative studies and those supporting causal links. For example, case–control and cohort studies have associated GSTM1 null genotypes with increased liver cancer risk in populations exposed to aflatoxins [269,270]. Conversely, functional studies confirm that the GSTM1 null genotype directly reduces detoxification capacity, providing mechanistic evidence for causality [271,272].

Similarly, polymorphisms in DNA repair genes such as XRCC1 and ERCC2 have been linked to differential susceptibility to UV- and chemical-induced cancers. Experimental assays demonstrate that specific XRCC1 variants impair base excision repair efficiency, causally increasing mutation rates upon toxin exposure [273,274,275].

Ignoring such genetic heterogeneity risks oversimplifying risk assessments and may leave vulnerable populations unprotected. Integrating both genetic screening and environmental exposure data is essential to identify high-risk groups and tailor preventative strategies effectively.

### 5.5. Global Disparities and Data Gaps in LMICs

Environmental carcinogen exposure is not distributed evenly across the globe [276]. Populations in low- and middle-income countries (LMICs) often face higher exposure levels due to weaker environmental regulations, inadequate monitoring, and unsafe agricultural or industrial practices. However, research from these regions is scarce, creating significant data gaps that limit global risk evaluations and exacerbate inequities in cancer prevention strategies [277].

Disparities are evident in exposure monitoring, with many LMICs lacking comprehensive biomonitoring programs compared to high-income countries (HICs) [278,279,280]. Similarly, research coverage is uneven: while extensive cohort studies and mechanistic investigations exist in North America and Europe, corresponding data from Africa, South Asia, and Latin America remain limited [281,282].

It is essential to distinguish between correlational and causal evidence. Some studies report associations between industrial emissions and increased cancer incidence in LMICs [283,284,285], but causality is often unconfirmed due to a lack of longitudinal or mechanistic data. Conversely, experimental studies and biomarker analyses in select populations provide more substantial evidence for causal links between specific exposures, such as aflatoxins or arsenic, and liver or skin cancers [286,287].

These disparities highlight the urgent need for improved monitoring systems, broader research coverage, and harmonized regulatory policies to reduce exposure inequities and better inform global cancer prevention strategies.

### 5.6. Contested Evidence and Bias in Risk Assessment

Epidemiological data on environmental carcinogens remain contentious. Confounding factors, methodological inconsistencies, and selective reporting contribute to divergent conclusions across studies [288]. In some cases, industry funding has raised concerns about bias in risk assessments, undermining public trust [289]. These controversies underscore the pressing need for transparent, independent research and standardized methodologies to enhance the reliability and policy relevance of findings.

Adding further complexity, metals illustrate a paradox in cancer research and treatment [290]. While certain metals, such as cadmium and arsenic, are recognized as carcinogenic, others form the basis of frontline chemotherapeutic agents [291]. For instance, platinum-based compounds like cisplatin and carboplatin exploit the cytotoxic potential of metals to induce DNA damage in tumor cells, serving as essential cancer treatments [292]. This dual role, where metals can act as both risk factors and therapeutic tools, demonstrates the importance of context when evaluating their health impacts [293].

Evidence for other environmental exposures is far less consistent. For example, studies on low-dose cadmium exposure report both significant associations with prostate and kidney cancers [294,295] and null findings in other cohorts [296,297], highlighting limitations due to small sample sizes, exposure misclassification, or regional differences. Importantly, regulatory agencies classify cadmium as a carcinogen only for inhalation exposure (as reflected in the slope factor established by USEPA, 1987, and OEHHA, 1987) [298,299], with insufficient evidence for carcinogenicity via ingestion. This underscores the need to consider exposure route, target organ specificity, and mechanistic plausibility when interpreting such findings. These factors are essential because a chemical’s carcinogenic potential cannot be fully understood without examining how it enters the body, which tissues it reaches at biologically meaningful concentrations, and whether its known molecular actions align with the observed cancer outcomes. For instance, agents classified as carcinogenic via inhalation may not exert similar effects when ingested, and epidemiological associations involving organs not typically targeted by a compound may signal confounding rather than true causation [300]. Integrating toxicokinetic data, mechanistic evidence, and exposure science therefore provides a more reliable framework for evaluating risks and distinguishing genuine causal relationships from statistical correlations arising from bias, co-exposures, or measurement limitations.

Moreover, the distinction between causal and correlational interpretations is critical. Biomarker and mechanistic studies support causal links for some exposures, such as aflatoxins and hepatocellular carcinoma [301,302], whereas many epidemiological correlations, particularly for low-level industrial pollutants, remain associative and potentially confounded [303,304]. Evidence for many other environmental contaminants, such as those encountered at low doses, remains largely associative and susceptible to confounding. This variability highlights the broader challenge of disentangling true causal effects from background noise in population-based studies. These divergent findings emphasize the need to acknowledge uncertainty, consider biases, and integrate multi-level evidence from mechanistic experiments to population studies when evaluating carcinogen risk. Non-threshold carcinogens such as aflatoxin B1, fumonisin B1, nitrosamines, and polycyclic aromatic hydrocarbons require the use of cancer slope factors (also called cancer potency factors) to quantitatively estimate risk. These slope factors are established by national and international agencies, including the US EPA IRIS, OEHHA, Health Canada, EFSA, and JECFA. Table S2 summarizes the available slope factors for the chemicals discussed in this review.

## 6. Innovations and Novel Approaches

While traditional toxicological studies have significantly advanced our understanding of how environmental toxins contribute to cancer, emerging research highlights the need for more precise, predictive and preventive tools. Conventional models often fall short in capturing the complexity of mixed exposures, inter-individual variability and long-term, low-dose effects [305]. To bridge these gaps, recent innovations are reshaping the field of environmental oncology. From biomarker discovery and advanced 3D organoid systems to CRISPR-based genetic screens and AI-driven predictive models, these novel approaches provide deeper mechanistic insights and open new avenues for prevention and intervention [306]. At the same time, the integration of public health monitoring systems and green chemistry solutions underscores a growing emphasis on translational impact, ensuring scientific advances contribute directly to reducing cancer risks linked to environmental toxins [307]. Emerging technologies are reshaping environmental oncology, from AI-based exposure prediction to organoid modeling and CRISPR screening for susceptibility genes (Table 4).

### 6.1. Biomarker Discovery for Toxin Exposure

Advances in molecular science have accelerated the discovery of biomarkers that can signal early exposure to carcinogenic toxins. Examples include DNA adducts, altered proteins, circulating microRNAs, and metabolite profiles that reveal biological changes long before tumors become clinically evident [327]. These biomarkers not only enhance the monitoring of environmental exposures but also hold promise for early diagnosis and personalized evaluation of cancer risk. Recent discoveries over the past decade include specific aflatoxin-DNA adducts as biomarkers of exposure and liver cancer risk, with mechanistic studies supporting causal links [328,329]. Circulating microRNAs such as miR-21 and miR-155 have emerged as indicators of exposure to polycyclic aromatic hydrocarbons (PAHs) and heavy metals, though many studies remain associative, linking elevated microRNA levels with exposure and early tumorigenesis [330,331,332].

**Figure 5:**
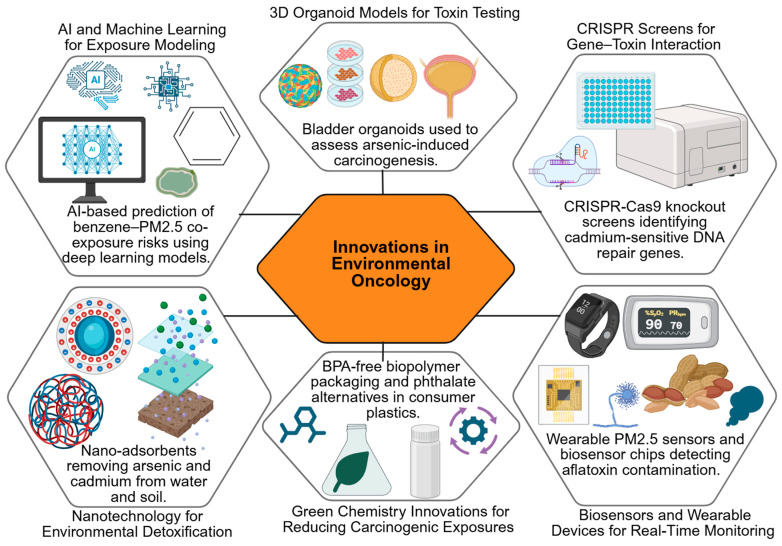
Innovations in Environmental Oncology. Created in BioRender. Busselberg, D. (2025) https://BioRender.com/b9ha324. accessed on 12 December 2025. Legend: AI—Artificial Intelligence, CRISPR—Clustered Regularly Interspaced Short Palindromic Repeats, PM2.5—Particulate Matter ≤ 2.5 µm, BPA—Bisphenol A.

Metabolomic profiling has identified altered levels of specific metabolites, such as urinary 8-oxo-dG and N-acetyl-S-(carbamoyl)methyl-cysteine, in populations exposed to industrial carcinogens. Some studies demonstrate causal mechanistic links between these metabolites and oxidative DNA damage [333,334], while others report correlations with exposure levels without confirmed causality [335,336]. These examples underscore the importance of integrating both mechanistic and population-based studies to distinguish biomarkers that are truly predictive of cancer risk from those that are merely indicative of exposure. Emerging tools and strategies in environmental oncology are summarized in a schematic (Figure 5) that captures the promise of AI, organoid models, CRISPR technologies, biosensors, and green chemistry approaches.

### 6.2. Organoids and 3D Models for Toxin Testing

Conventional two-dimensional cell cultures provide limited insights into how toxins act in the human body [337]. The development of organoids and three-dimensional culture systems has transformed experimental toxicology by recreating organ-like structures with realistic cell diversity and architecture [338]. These platforms enable more accurate testing of toxin-induced carcinogenesis, bridging the gap between laboratory models and human responses.

Over the past decade, practical applications of organoid models have expanded significantly. For example, liver organoids have been used to study aflatoxin B1-induced hepatocellular carcinoma, providing causal evidence of DNA adduct formation and mutational signatures [339,340,341]. Similarly, kidney and bladder organoids have been used to assess cadmium and arsenic toxicity, revealing mechanisms such as oxidative stress, apoptosis, and epigenetic alterations [342,343,344]. Gut and colon organoids have provided insights into polycyclic aromatic hydrocarbon and nitrosamine exposure, demonstrating how these toxins induce inflammation and DNA damage in tissue-specific contexts [345,346,347]. These studies highlight the utility of 3D models for mechanistic investigations that are difficult to replicate in 2D cultures or animal models. Organoid and 3D culture platforms offer a versatile and physiologically relevant system for understanding human-specific responses to environmental carcinogens, enabling both causal inference and high-throughput screening of potential protective interventions.

### 6.3. CRISPR Screens for Susceptibility Genes

The application of CRISPR-Cas9 technology has opened new avenues for exploring genetic susceptibility to toxins [348]. High-throughput CRISPR screens can identify genes and pathways that determine how cells respond to carcinogenic exposures, whether by influencing DNA repair, detoxification, or survival [349]. Such studies not only clarify mechanisms of vulnerability but also highlight potential molecular targets for intervention. Recent examples include CRISPR screens that identified genes such as TP53, BRCA1, and XRCC1 as modulators of the cellular response to aflatoxin and benzene exposure. Functional knockout of these genes in human liver and hematopoietic cell lines demonstrated causal increases in DNA damage and mutagenesis, confirming their role in toxin susceptibility [350,351]. Other CRISPR studies have identified candidate susceptibility loci in detoxification pathways, including GSTM1, CYP1A1, and NQO1. While some screens show direct mechanistic links to increased carcinogen-induced cytotoxicity [352,353,354], others report associative findings where gene perturbations correlate with altered stress responses without definitive causal proof [355,356]. These CRISPR-based approaches thus provide powerful tools to distinguish causal genetic determinants of toxin susceptibility from associative markers, enabling targeted interventions and improved risk stratification in exposed populations.

### 6.4. AI and Predictive Models for Exposure–Outcome Relationships

Artificial intelligence and machine learning are increasingly being used to analyze complex toxicological data [357]. By integrating information from epidemiological studies, molecular assays, and environmental monitoring, AI-driven models can predict cancer risks linked to low-dose or mixed exposures [358]. These tools enable more nuanced risk assessments, providing decision-makers with evidence-based insights for informed regulatory policies.

Recent applications include AI models that predict hepatocellular carcinoma risk from aflatoxin exposure by integrating dietary, genetic, and biomarker data, achieving high predictive accuracy in population studies [359,360]. Machine learning has also been applied to multi-chemical exposure datasets to identify previously unrecognized interactions between heavy metals, pesticides, and airborne pollutants, providing mechanistic insights into combined carcinogenic effects [361,362,363]. Success stories include the use of deep learning to integrate organoid and 3D culture experimental data with population-level exposure records, enabling early identification of high-risk individuals and prioritization of protective interventions [364,365,366]. Overall, AI and predictive modeling offer a transformative approach to environmental oncology, enabling the integration of diverse datasets, the identification of complex exposure–response relationships, and enhanced decision-making for cancer prevention strategies.

### 6.5. Public Health Monitoring Innovations

Digital technologies are reshaping how environmental risks are tracked [367]. Wearable sensors, mobile applications, and geospatial data systems now enable real-time monitoring of exposures at both the individual and community levels [368]. When combined with large-scale health databases, these innovations help public health systems identify emerging risks earlier and implement targeted preventive measures.

Recent examples include population-level AI platforms that integrate air pollution data with electronic health records to predict cancer risk clusters, enabling proactive interventions in high-exposure regions [369,370,371]. Digital biomonitoring programs, such as wearable heavy metal sensors and smartphone-based pesticide tracking applications, have successfully demonstrated real-time exposure assessment in rural and industrial communities, providing actionable data for both public health authorities and individuals [372,373,374]. Geospatial surveillance systems combined with machine learning have also been used to map exposure hotspots for aflatoxins and airborne particulates, supporting targeted regulatory inspections and community education initiatives [375,376,377]. Together, these innovations illustrate the transformative potential of digital and AI-assisted monitoring to enhance environmental health surveillance and improve cancer prevention strategies globally.

### 6.6. Green Chemistry and Safer Consumer Alternatives

Preventive innovation is also advancing through green chemistry, which emphasizes the design of safer chemicals and processes to replace hazardous ones [378]. Toxin-free alternatives are increasingly being introduced into food packaging, cosmetics, agricultural products, and household materials, thereby reducing everyday exposure [379]. These strategies not only support cancer prevention but also align with sustainability goals and corporate responsibility. Recent innovations include the development of biodegradable, non-toxic pesticide formulations that reduce crop contamination without compromising yield, as demonstrated in several field trials across Asia and Africa [380,381,382]. Similarly, industrial chemical replacements, such as non-phthalate plasticizers and safer flame retardants, have been successfully implemented in consumer goods to minimize carcinogenic risks [383,384].

An essential priority within this framework is the substitution of carcinogenic metals with safer alternatives in both industrial applications and healthcare [385]. Heavy metals such as cadmium, lead, and chromium are still widely used in manufacturing processes despite their established toxicological risks [386]. Green chemistry approaches advocate for non-toxic or less hazardous replacements that can deliver the same functional benefits without compromising human or environmental health [387]. For example, zinc- and magnesium-based compounds are increasingly replacing cadmium in batteries and coatings, reducing occupational and environmental exposures [388,389]. In healthcare, research on metal-based drugs is exploring platinum derivatives with reduced toxicity and biodegradable metal–organic frameworks for drug delivery, exemplifying safer alternatives while maintaining therapeutic efficacy [390,391,392].

## 7. Challenges, Limitations, and Future Work

### 7.1. Challenges

Studying the links between environmental toxins and cancer presents several persistent challenges. A major concern is the absence of unified regulatory standards across regions, which makes it difficult to establish consistent risk assessments and exposure limits [393]. Another obstacle is the shortage of long-term exposure data, as most studies focus on short durations rather than tracking individuals over decades, a crucial factor given the long latency of many cancers [394]. In addition, real-world scenarios involve exposure to multiple toxins simultaneously, and understanding the combined or synergistic effects of such mixtures remains a complex and underexplored area [395].

Additional challenges include an incomplete understanding of the molecular mechanisms underlying toxin-induced carcinogenesis, particularly for low-dose or chronic exposures. For many environmental chemicals, the pathways linking exposure to DNA damage, epigenetic alterations, immune dysregulation, and tumor initiation remain only partially characterized [396,397,398]. Exposure assessment limitations further complicate research. Traditional methods relying on self-reported exposure or single-time-point biomonitoring may not accurately reflect cumulative or fluctuating exposures over time. Emerging approaches such as wearable sensors and metabolomic profiling offer improvements but are still limited in scope and accessibility, particularly in low- and middle-income countries [399,400,401]. Together, these gaps underscore the need for mechanistic studies, improved longitudinal exposure assessment, and standardized methodologies to strengthen causal inference in environmental carcinogen research.

### 7.2. Limitations of the Review

This review has certain limitations that should be acknowledged. The inclusion of only English-language publications may have excluded valuable research from non-English sources, potentially narrowing the scope of evidence. Moreover, as a secondary analysis, the review relies entirely on published data and does not contribute original experimental findings, leaving it subject to the strengths and weaknesses of existing literature. Additional limitations include potential simplifications in synthesizing complex mechanisms of toxin-induced carcinogenesis. By summarizing multifaceted biological pathways and diverse exposure scenarios, some nuances may be underrepresented, potentially oversimplifying risk interpretations [402,403]. Study selection bias is another concern. Despite systematic search strategies, certain high-quality studies may have been inadvertently omitted due to publication bias, database coverage, or focus on specific populations. This could influence conclusions regarding exposure–cancer relationships and generalizability across regions [404,405]. Acknowledging these limitations underscores the need for cautious interpretation and highlights areas where further primary research is warranted.

### 7.3. Future Work

Several research directions could help overcome current gaps. Multi-omics approaches that integrate genomic, transcriptomic, proteomic, epigenomic, and metabolomic data hold promise for unraveling how toxins influence cancer development at multiple biological levels [406]. Expanding community-level monitoring, through wearable devices, environmental mapping and citizen science initiatives, would provide more realistic assessments of exposure [407]. Building collaborative environmental cancer registries could enhance data sharing and allow for stronger global epidemiological studies [408]. Greater attention should also be given to mechanistic research on low-dose, long-term exposures, which are more representative of daily life than high-dose laboratory models [409]. Ultimately, future progress depends on closer cross-disciplinary collaboration, which brings together toxicology, oncology, environmental science, and public health to design integrated strategies for prevention, detection, and intervention.

Additional future directions include prioritizing research on vulnerable populations, such as genetically susceptible individuals or communities with high environmental exposure, to tailor preventive strategies more effectively [410,411,412]. There is also a critical need for the development of standardized, validated biomarkers and predictive models that can reliably translate mechanistic insights into practical policy interventions [413,414,415]. These tools could inform exposure limits, regulatory guidelines, and public health recommendations. Finally, integrating economic, behavioral, and policy research with molecular and epidemiological studies can help identify feasible interventions, optimize resource allocation, and ensure that scientific advances lead to tangible reductions in toxin-related cancer risk worldwide [416,417].

## 8. Conclusions

Environmental toxins are a significant yet frequently underestimated cause of cancer, acting through mechanisms such as oxidative stress, DNA damage, epigenetic changes, hormonal disruption, and modifications of the tumor microenvironment. Evidence from exposure studies on benzene, aflatoxins, arsenic, phthalates, particulate matter, and microplastics highlights their real-world impact on human health. Recent advances, such as biomarker discovery, organoid and 3D model testing, CRISPR-based genetic screens, and AI-driven predictive modeling, are transforming research and improving our ability to assess and mitigate these risks. Moving forward, progress will require integrated strategies that combine mechanistic understanding, advanced exposure monitoring, longitudinal studies, and predictive modeling, supported by close collaboration across oncology, toxicology, and environmental sciences. Ensuring global research equity is equally vital, so that populations in low- and middle-income countries, which often face higher environmental exposures, are fully represented in data, policy decisions, and intervention efforts. Together, these approaches can strengthen prevention efforts, inform policy, and reduce the burden of toxin-related cancers worldwide.

## Figures and Tables

**Table 1:** Mechanisms of Oxidative Stress–Mediated Carcinogenesis.

Environmental Toxin	ROS Source/Mechanism	DNA Lesions Induced	Key Pathways Affected	Tumor Suppressor Impact	Cancer Association	Evidence Type (In Vitro/In Vivo/Epidemiology)
Arsenic	Mitochondrial dysfunction	8-oxoG	NF-κB, MAPK	p53 inhibition	Skin, bladder cancer	In vitro + human cohort [29]
Cadmium	Redox cycling	SSBs, DSBs	PI3K/AKT	BRCA1 suppression	Lung cancer	Animal studies [30]
Benzene	CYP2E1 metabolism	Chromosomal breaks	STAT3	p53 mutation	Leukemia	Epidemiology [31]
Chromium (VI)	Fenton reaction	DNA crosslinks	ERK/MAPK	ATM/ATR inhibition	Lung cancer	In vitro [32]
Aflatoxin B1	CYP450 activation	DNA adducts	Wnt/β-catenin	p53 codon 249 mutation	HCC	Case-control [33]
PM2.5	Mitochondrial ROS	Oxidative DNA lesions	Nrf2	p16 silencing	Lung cancer	Human exposure [34]
Nickel	ROS + histone binding	DNA-protein crosslinks	HIF-1α	p53 silencing	Nasopharyngeal cancer	In vitro [35]
Pesticides (DDT)	Lipid peroxidation	DNA fragmentation	AKT	Bax suppression	Breast cancer	Epidemiology [36]

**Table 2:** Epigenetic Reprogramming Induced by Environmental Toxins: Mechanisms, Evidence Type, and Cancer Relevance.

Toxin/Compound	DNA Methylation Effects (Mechanism)	Histone Modifications (Mechanism)	miRNA Dysregulation	Genes/Pathways Affected	Cancer Link	Evidence Type	References
Arsenic	Hypermethylation of tumor suppressor promoters via DNMT1 overactivation	H3K9 acetylation loss leading to chromatin condensation	↑miR-21, ↓miR-200b	p16, MLH1, E-cadherin	Bladder, skin, lung	Causal (human & in vivo)	[49,50,51]
Cadmium	Global DNA hypomethylation due to DNMT inhibition	Altered H3K27me3 represses antioxidant genes	↓miR-143, ↑miR-21	GSTP1, MT1A	Prostate, liver	Causal (animal)	[52,53,54]
Bisphenol A (BPA)	CpG methylation of ERα promoter affecting estrogen signaling	Histone acetylation at ESR1 and HOXC6 loci	↑miR-146a, ↓miR-29	ESR1, BRCA1	Breast, endometrial	Correlational (epidemiological)	[55,56,57]
Nickel	Promoter hypermethylation through ROS-mediated DNMT1 activation	Inhibition of H3K4me2 causes transcriptional silencing	↑miR-222, ↓miR-152	E-cadherin, CDH1	Lung, nasal	Causal (in vitro)	[58]
Aflatoxin B1	p53 codon 249 mutation with CpG methylation interference	H4 hypoacetylation impairs DNA repair	↓miR-122, ↑miR-34a	TP53, CYP450	Liver (HCC)	Causal (human + animal)	[59,60]
Airborne PM2.5	LINE-1 hypomethylation through oxidative stress-induced TET activation	H3K9me3 alterations affecting chromatin structure	↑miR-210, ↑miR-222	DNA repair, oxidative stress genes	Lung, colorectal	Correlational (cohort)	[61,62,63]
Phthalates (DEHP, DBP)	Demethylation of oncogene promoters via DNMT suppression	Disruption of histone acetyltransferase (HAT) activity	↑miR-10b, ↑miR-21	MMPs, BCL2	Breast, testicular	Causal (animal)	[64,65,66]
Chromium (VI)	CpG hypermethylation through oxidative stress–DNMT coupling	H3K27me3 enrichment silences CDKN2A	↓miR-200c, ↓miR-143	CDKN2A, MLH1	Lung	Causal (in vitro + animal)	[67,68,69]

Key:↑ = upregulated, ↓ = downregulated, DNMT = DNA methyltransferase; HAT = histone acetyltransferase; TET = ten-eleven translocation enzyme, Causal evidence: Direct mechanistic proof in controlled in vitro/in vivo models, Correlational evidence: Associations observed in population/epidemiological studies.

**Table 3:** Case Studies of Specific Environmental Toxins, Exposure Routes, and Associated Cancers.

Toxin/Compound	Exposure Route(s)	Bioactivation/Metabolic Pathway	Key Molecular Targets	Hallmark Cancer Mechanism	Cancer Outcome	Region Most Studied	References
Benzene	Inhalation (industrial, vehicular emissions), percutaneous (solvent handling)	CYP2E1 → benzene oxide → phenol/catechol metabolites	Bone marrow stem cells, topoisomerase II	Chromosomal aberrations, oxidative DNA damage	Acute myeloid leukemia (AML), lymphoma	USA, China	[131,132,133]
Aflatoxin B1	Ingestion (contaminated grains, nuts)	CYP450 → AFB1-8,9-epoxide → DNA adducts	p53 codon 249, GST	Mutagenesis, impaired DNA repair	Hepatocellular carcinoma	Africa, Asia	[134,135,136]
Arsenic	Ingestion (contaminated water, rice), inhalation (smelting), percutaneous (soil)	Methylation to MMA/DMA by AS3MT	Keratinocytes, endothelial cells	ROS production, epigenetic remodeling	Skin, bladder, lung cancer	Bangladesh, Taiwan	[137,138,139]
Phthalates (DEHP, DBP)	Ingestion (food packaging), inhalation (dust), percutaneous (cosmetics)	Hydrolysis to monoesters (MEHP, MBP)	ER/AR receptors, peroxisome proliferator pathways	Endocrine disruption, hormone mimicry	Breast, testicular, prostate cancer	Global	[140,141]
PM2.5 (Fine Particulate Matter)	Inhalation (ambient air pollution)	Mitochondrial ROS generation, inflammation	Lung epithelial cells, DNA repair enzymes	Oxidative stress, genomic instability	Lung, colorectal cancer	China, Europe	[142,143,144]
Microplastics	Ingestion (seafood, bottled water), inhalation (indoor air)	Additive leaching, oxidative degradation	Gut microbiota, epithelial barrier	Chronic inflammation, dysbiosis	Colorectal, hepatic cancer risk	Emerging (Asia, Europe)	[145,146,147]
Chromium (VI)	Occupational inhalation, dermal absorption	Reduction to Cr(V)/Cr(III) intermediates	DNA repair enzymes, histones	DNA–protein crosslinks, ROS formation	Lung, nasal cancer	Industrial workers (USA, China)	[148,149,150]
Pesticides (DDT, organophosphates, glyphosate)	Ingestion (food residues), inhalation (aerosol drift), percutaneous (farm handling)	Bioaccumulation and CYP-mediated activation	Estrogen receptor, AChE enzyme, oxidative stress pathways	Endocrine disruption, genotoxicity, oxidative damage	Breast, prostate, non-Hodgkin lymphoma	Africa, Latin America, India	[151,152,153]

**Table 4:** Innovations in Environmental Oncology: Mechanistic Advances, Applications, and Limitations.

Innovation	Application	Mechanistic Advantage	Example Use Case	Research Stage	Benefits	Limitations	References
AI Models	Exposure prediction, risk modeling	Simulates multi-exposure interactions and cancer risk pathways	Machine learning model integrating benzene + PM2.5 to predict leukemia risk	Advanced	Enables early detection and predictive toxicology	Data bias, need for diverse training datasets	[308,309,310]
Organoids	3D human cancer models	Replicates tissue microenvironment for toxin testing	Human bladder organoids for arsenic-induced carcinogenesis	Preclinical	Human relevance, ethical alternative to animal models	High cost, standardization issues	[311,312,313]
CRISPR Screens	Mapping gene-toxin susceptibility	Identifies genetic pathways mediating toxin response	CRISPR-Cas9 knockout of BRCA1 to study cadmium-induced DNA damage	Experimental	Enables precision medicine and targeted prevention	Off-target edits, ethical issues	[314]
Biosensors	Real-time environmental toxin monitoring	High sensitivity and specificity for on-site toxin detection	Nanobiosensor detecting aflatoxin B1 in maize	Applied	Early detection and public health prevention	Requires frequent calibration, cost in LMICs	[315,316,317]
Wearables	Personal exposure tracking	Continuous, individualized monitoring of pollutant exposure	PM2.5-tracking smart badge used in urban cancer risk studies	Pilot	Enables population-level exposure mapping	Accessibility, data privacy	[318,319]
Green Chemistry	Substitution of carcinogenic compounds	Reduces formation of reactive intermediates	Development of BPA-free plastics and phthalate alternatives	Applied	Preventive and sustainable	Slow industry adoption, cost barriers	[320]
Multi-omics	Mechanistic profiling of toxin response	Integrates genomics, transcriptomics, metabolomics, and epigenomics	Multi-omics study on microplastics–gut microbiota–colon cancer link	Experimental	Comprehensive mechanistic insight	Complex data integration	[321,322,323]
Environmental Registries	Data surveillance and trend mapping	Tracks long-term toxin exposure–cancer correlations globally	Global arsenic and cadmium exposure registries	Ongoing	Supports epidemiological tracking	Data gaps in LMICs	[324,325,326]

## Data Availability

No new data were created or analyzed in this study. Data sharing is not applicable to this article.

## Supplementary Materials

The following supporting information can be downloaded at: https://www.mdpi.com/article/10.3390/jox16010002/s1, Table S1: IARC Classification of Key Environmental Carcinogens Included in This Review; Table S2: Toxicological Reference Values (Slope Factors) for Known and Probable Human Carcinogens: National and International Guidelines. References [299,418,419,420,421,422] are cited in the supplementary materials.

## Abbreviations

The following abbreviations are used in this manuscript:

3D	Three-Dimensional
AI-driven	Artificial Intelligence–driven
AKT	Protein Kinase B (cell survival signaling)
AML	Acute Myeloid Leukemia
ATM/ATR	Ataxia Telangiectasia Mutated/Rad3-related protein kinases
BRCA1	Breast Cancer Gene 1
CDKN2A	Cyclin-Dependent Kinase Inhibitor 2A
CpG	Cytosine–Phosphate–Guanine sites in DNA
CRISPR	Clustered Regularly Interspaced Short Palindromic Repeats
Cr(V)	Hexavalent Chromium (Valence V)
CYP2E1	Cytochrome P450 Family 2 Subfamily E Member 1
CYP450	Cytochrome P450 Enzymes
DDT	Dichlorodiphenyltrichloroethane (pesticide)
DNA	Deoxyribonucleic Acid
↓miR-143	Downregulation of microRNA-143
E-cadherin	Epithelial cadherin (cell adhesion protein)
EDCs	Endocrine-Disrupting Chemicals
ERK/MAPK	Extracellular Signal-Regulated Kinase/MAPK pathway
ESR1	Estrogen Receptor 1
G→T	Guanine to Thymine Mutation
GSTP1	Glutathione S-transferase Pi 1
H3K27me3	Trimethylation of lysine 27 on histone H3
HCC	Hepatocellular Carcinoma
Hg	Mercury
HIF-1α	Hypoxia-Inducible Factor 1-alpha
IL-6, TNF-α, IL-1β	Inflammatory Cytokines (Interleukin-6, Tumor Necrosis Factor-alpha, Interleukin-1 beta)
LINE-1	Long Interspersed Nuclear Element-1
LMICs	Low- and Middle-Income Countries
MAPK	Mitogen-Activated Protein Kinase
miRNA	MicroRNA
MLH1	MutL Homolog 1 (DNA mismatch repair gene)
MMA/DMA	Monomethylarsonic Acid/Dimethylarsinic Acid
M2	M2-polarized Macrophages
MeSH	Medical Subject Headings
NF-κB	Nuclear Factor kappa-light-chain-enhancer of activated B cells
Nrf2	Nuclear factor erythroid 2–related factor 2
Pb	Lead
PI3K/AKT	Phosphoinositide 3-Kinase/Protein Kinase B pathway
PM2.5	Particulate Matter ≤ 2.5 μm
PubMed	Public/Publisher MEDLINE (Biomedical Literature Database)
p16	Cyclin-Dependent Kinase Inhibitor 2A (CDKN2A)
p53	Tumor Protein p53 (Guardian of the Genome)
RNS	Reactive Nitrogen Species
ROS	Reactive Oxygen Species
STAT3	Signal Transducer and Activator of Transcription 3
TME	Tumor Microenvironment
TP53	Tumor Protein 53 (p53 gene)
VEGF	Vascular Endothelial Growth Factor
Wnt/β-catenin	Wnt Signaling Pathway with β-catenin involvement
↑miR-146a	Upregulation of microRNA-146a
↑miR-210	Upregulation of microRNA-210
↑miR-222	Upregulation of microRNA-222
C→A	Cytosine to Adenine Mutation

## References

[1] Bray F., Laversanne M., Sung H., Ferlay J., Siegel R.L., Soerjomataram I., Jemal A. (2024). Global cancer statistics 2022: GLOBOCAN estimates of incidence and mortality worldwide for 36 cancers in 185 countries. CA Cancer J. Clin..

[2] Yuan S., Almagro J., Fuchs E. (2024). Beyond genetics: Driving cancer with the tumour microenvironment behind the wheel. Nat. Rev. Cancer.

[3] Wu X., Zhang X., Yu X., Liang H., Tang S., Wang Y. (2025). Exploring the association between air pollution and the incidence of liver cancers. Ecotoxicol. Environ. Saf..

[4] Tang F.R. (2024). Health Effect of Low-Dose-Rate Irradiation with Cumulative Threshold Dose: A Promising Area to Explore in Nuclear Emergency and Environmental Contamination. Cells.

[5] Rizeq B., Gupta I., Ilesanmi J., AlSafran M., Rahman M.M., Ouhtit A. (2020). The Power of Phytochemicals Combination in Cancer Chemoprevention. J. Cancer.

[6] Santofimio V.Q., Amaral A.F.S., Feary J. (2024). Occupational exposures in low- and middle-income countries: A scoping review. PLoS Glob. Public Health.

[7] Fuller R., Landrigan P.J., Balakrishnan K., Bathan G., Bose-O’Reilly S., Brauer M., Caravanos J., Chiles T., Cohen A., Corra L. (2022). Pollution and health: A progress update. Planet. Health.

[8] Debela D.T., Muzazu S.G., Heraro K.D., Ndalama M.T., Mesele B.W., Haile D.C., Kitui S.K., Manyazewal T. (2021). New approaches and procedures for cancer treatment: Current perspectives. SAGE Open Med..

[9] Braillon A., Lang A.E. (2023). The International Agency for Research on Cancer and e-cigarette carcinogenicity: Time for an evaluation. Eur. J. Epidemiol..

[10] https://www.who.int/news-room/fact-sheets/detail/cancer.

[11] Lagoa R., Marques-da-Silva D., Diniz M., Daglia M., Bishayee A. (2022). Molecular mechanisms linking environmental toxicants to cancer development: Significance for protective interventions with polyphenols. Semin. Cancer Biol..

[12] Wang M., Xiao Y., Miao J., Zhang X., Liu M., Zhu L., Liu H., Shen X., Wang J., Xie B. (2025). Oxidative Stress and Inflammation: Drivers of Tumorigenesis and Therapeutic Opportunities. Antioxidants.

[13] Xiao Q., Liu Y., Li T., Wang C., He S., Zhai L., Yang Z., Zhang X., Wu Y., Liu Y. (2025). Viral oncogenesis in cancer: From mechanisms to therapeutics. Signal Transduct. Target. Ther..

[14] Bonfiglio R., Sisto R., Casciardi S., Palumbo V., Scioli M.P., Palumbo A., Trivigno D., Giacobbi E., Servadei F., Melino G. (2024). The impact of toxic metal bioaccumulation on colorectal cancer: Unravelling the unexplored connection. Sci. Total Environ..

[15] Mandal M., Sarkar M., Khan A., Biswas M., Masi A., Rakwal R., Agrawal G.K., Srivastava A., Sarkar A. (2022). Reactive Oxygen Species (ROS) and Reactive Nitrogen Species (RNS) in plants– maintenance of structural individuality and functional blend. Adv. Redox Res..

[16] Krishnamurthy H.K., Pereira M., Rajavelu I., Jayaraman V., Krishna K., Wang T., Bei K., Rajasekaran J.J. (2024). Oxidative stress: Fundamentals and advances in quantification techniques. Front. Chem..

[17] Chatterjee N., Walker G.C. (2017). Mechanisms of DNA damage, repair, and mutagenesis. Environ. Mol. Mutagen..

[18] Markkanen E. (2017). Not breathing is not an option: How to deal with oxidative DNA damage. DNA Repair.

[19] Siametis A., Garinis G.A. (2025). From Genome to Geroscience: How DNA Damage Shapes Systemic Decline. BioEssays.

[20] Ma N., Wang Y., Li X., Xu M., Tan D. (2025). Reactive oxygen species in cancer: Mechanistic insights and therapeutic innovations. Cell Stress Chaperones.

[21] Kowalczyk P., Sulejczak D., Kleczkowska P., Bukowska-Ośko I., Kucia M., Popiel M., Wietrak E., Kramkowski K., Wrzosek K., Kaczyńska K. (2021). Mitochondrial Oxidative Stress—A Causative Factor and Therapeutic Target in Many Diseases. Int. J. Mol. Sci..

[22] Tiwari V., Wilson D.M. (2019). DNA Damage and Associated DNA Repair Defects in Disease and Premature Aging. Am. J. Hum. Genet..

[23] Huang R., Zhou P.-K. (2021). DNA damage repair: Historical perspectives, mechanistic pathways and clinical translation for targeted cancer therapy. Signal Transduct. Target. Ther..

[24] Venkatesan S., Natarajan A.T., Hande M.P. (2015). Chromosomal instability—Mechanisms and consequences. Mutat. Res. Toxicol. Environ. Mutagen..

[25] Li Q., Geng S., Luo H., Wang W., Mo Y.-Q., Luo Q., Wang L., Song G.-B., Sheng J.-P., Xu B. (2024). Signaling pathways involved in colorectal cancer: Pathogenesis and targeted therapy. Signal Transduct. Target. Ther..

[26] Jalal S., Earley J.N., Turchi J.J. (2011). DNA Repair: From Genome Maintenance to Biomarker and Therapeutic Target. Clin. Cancer Res..

[27] Sharifi-Rad M., Kumar N.V.A., Zucca P., Varoni E.M., Dini L., Panzarini E., Rajkovic J., Fokou P.V.T., Azzini E., Peluso I. (2020). Lifestyle, Oxidative Stress and Antioxidants: Back and Forth in the Pathophysiology of Chronic Diseases. Front. Physiol..

[28] Klapacz J., Pottenger L.H., Engelward B.P., Heinen C.D., Johnson G.E., Clewell R.A., Carmichael P.L., Adeleye Y., Andersen M.E. (2016). Contributions of DNA repair and damage response pathways to the non-linear genotoxic responses of alkylating agents. Mutat. Res. Mutat. Res..

[29] Speer R.M., Zhou X., Volk L.B., Liu K.J., Hudson L.G. (2023). Arsenic and cancer: Evidence and mechanisms. Adv. Pharmacol..

[30] Kulkarni P., Dasgupta P., Bhat N.S., Hashimoto Y., Saini S., Shahryari V., Yamamura S., Shiina M., Tanaka Y., Dahiya R. (2020). Role of the PI3K/Akt pathway in cadmium induced malignant transformation of normal prostate epithelial cells. Toxicol. Appl. Pharmacol..

[31] Ramírez-Lopera V., Uribe-Castro D., Bautista-Amorocho H., Silva-Sayago J.A., Mateus-Sánchez E., Ardila-Barbosa W.Y., Pérez-Cala T.L. (2021). The effects of genetic polymorphisms on benzene-exposed workers: A systematic review. Health Sci. Rep..

[32] Chen Q.Y., Murphy A., Sun H., Costa M. (2019). Molecular and epigenetic mechanisms of Cr(VI)-induced carcinogenesis. Toxicol. Appl. Pharmacol..

[33] Moreno-León C., Aguayo F. (2025). Cooperation Between Aflatoxin-Induced p53 Aberrations and Hepatitis B Virus in Hepatocellular Carcinoma. J. Xenobiotics.

[34] Vilas-Boas V., Chatterjee N., Carvalho A., Alfaro-Moreno E. (2024). Particulate matter-induced oxidative stress—Mechanistic insights and antioxidant approaches reported in in vitro studies. Environ. Toxicol. Pharmacol..

[35] Huang R.-X., Zhou P.-K. (2020). DNA damage response signaling pathways and targets for radiotherapy sensitization in cancer. Signal Transduct. Target. Ther..

[36] Subah Z., Ryu J.H. (2024). Impact of DDT on women’s health in Bangladesh: Escalating breast cancer risk and disturbing menstrual cycle. Front. Public Health.

[37] Marczylo E.L., Jacobs M.N., Gant T.W. (2016). Environmentally induced epigenetic toxicity: Potential public health concerns. Crit. Rev. Toxicol..

[38] Manić L., Wallace D., Onganer P.U., Taalab Y.M., Farooqi A.A., Antonijević B., Djordjevic A.B. (2022). Epigenetic mechanisms in metal carcinogenesis. Toxicol. Rep..

[39] Liu P., Yang F., Zhang L., Hu Y., Chen B., Wang J., Su L., Wu M., Chen W. (2022). Emerging role of different DNA methyltransferases in the pathogenesis of cancer. Front. Pharmacol..

[40] Aanniz T., Bouyahya A., Balahbib A., Kadri K.E., Khalid A., Makeen H.A., Alhazmi H.A., Omari N.E., Zaid Y., Wong R.S.-Y. (2024). Natural bioactive compounds targeting DNA methyltransferase enzymes in cancer: Mechanisms insights and efficiencies. Chem. Biol. Interact..

[41] Salmerón-Bárcenas E.G., Martínez-Zayas A., Vargas-Mejía M., Villegas-Sepúlveda N., Briseño-Díaz P., Aguilar-Rojas A., Baños-Hernández C.J., Torres-Rojas F.I., Antaño-Arias R., Hernández-Rivas R. (2025). DNMT Enzymes and Their Impact on Cervical Cancer: A State-of-the-Art Review. Int. J. Mol. Sci..

[42] Prabhu K.S., Sadida H.Q., Kuttikrishnan S., Junejo K., Bhat A.A., Uddin S. (2024). Beyond genetics: Exploring the role of epigenetic alterations in breast cancer. Pathol.-Res. Pract..

[43] Reddy S.R., Bangeppagari M., Lee S.J. (2025). Immune–Epigenetic Effects of Environmental Pollutants: Mechanisms, Biomarkers, and Transgenerational Impact. Curr. Issues Mol. Biol..

[44] Sobral A.F., Cunha A., Costa I., Silva-Carvalho M., Silva R., Barbosa D.J. (2025). Environmental Xenobiotics and Epigenetic Modifications: Implications for Human Health and Disease. J. Xenobiotics.

[45] Kurita H., Ohuchi K., Inden M. (2025). Effects of Environmental Non-Essential Toxic Heavy Metals on Epigenetics During Development. Toxics.

[46] Chaiwangyen W., Khantamat O., Kangwan N., Tipsuwan W., de Sousa F.L.P. (2025). MicroRNA expression in response to environmental hazards: Implications for health. Ecotoxicol. Environ. Saf..

[47] Hammouz R.Y., Kołat D., Kałuzińska Ż., Płuciennik E., Bednarek A.K. (2021). MicroRNAs: Their Role in Metastasis, Angiogenesis, and the Potential for Biomarker Utility in Bladder Carcinomas. Cancers.

[48] Aslam M.I., Patel M., Singh B., Jameson J.S., Pringle J.H. (2012). MicroRNA manipulation in colorectal cancer cells: From laboratory to clinical application. J. Transl. Med..

[49] Lu G., Xu H., Chang D., Wu Z., Yao X., Zhang S., Li Z., Bai J., Cai Q., Zhang W. (2014). Arsenic exposure is associated with DNA hypermethylation of the tumor suppressor gene p16. J. Occup. Med. Toxicol..

[50] Liu B., Song J., Luan J., Sun X., Bai J., Wang H., Li A., Zhang L., Feng X., Du Z. (2016). Promoter methylation status of tumor suppressor genes and inhibition of expression of DNA methyltransferase 1 in non-small cell lung cancer. Exp. Biol. Med..

[51] Lee H., Zhang P., Herrmann A., Yang C., Xin H., Wang Z., Hoon D.S.B., Forman S.J., Jove R., Riggs A.D. (2012). Acetylated STAT3 is crucial for methylation of tumor-suppressor gene promoters and inhibition by resveratrol results in demethylation. Proc. Natl. Acad. Sci. USA.

[52] Ren C., Ren L., Yan J., Bai Z., Zhang L., Zhang H., Xie Y., Li X. (2021). Cadmium causes hepatopathy by changing the status of DNA methylation in the metabolic pathway. Toxicol. Lett..

[53] Geng H., An Q., Song J., He D., Han H., Wang L. (2024). Cadmium-induced global DNA hypermethylation promoting mitochondrial dynamics dysregulation in hippocampal neurons. Environ. Toxicol..

[54] Liu Y., Sun Y., Yang J., Wu D., Yu S., Liu J., Hu T., Luo J., Zhou H. (2024). DNMT1-targeting remodeling global DNA hypomethylation for enhanced tumor suppression and circumvented toxicity in oral squamous cell carcinoma. Mol. Cancer.

[55] Bhandari R.K., Taylor J.A., Sommerfeld-Sager J., Tillitt D.E., Ricke W.A., vom Saal F.S. (2019). Estrogen receptor 1 expression and methylation of Esr1 promoter in mouse fetal prostate mesenchymal cells induced by gestational exposure to bisphenol A or ethinylestradiol. Environ. Epigenet..

[56] Stanojević M., Dolenc M.S. (2025). Mechanisms of bisphenol A and its analogs as endocrine disruptors via nuclear receptors and related signaling pathways. Arch. Toxicol..

[57] Qin T., Zhang X., Guo T., Yang T., Gao Y., Hao W., Xiao X. (2021). Epigenetic Alteration Shaped by the Environmental Chemical Bisphenol A. Front. Genet..

[58] Zhao Y., Fan X., Wang Q., Zhen J., Li X., Zhou P., Lang Y., Sheng Q., Zhang T., Huang T. (2023). ROS promote hyper-methylation of NDRG2 promoters in a DNMTS-dependent manner: Contributes to the progression of renal fibrosis. Redox Biol..

[59] Chittmittrapap S., Chieochansin T., Chaiteerakij R., Treeprasertsuk S., Klaikaew N., Tangkijvanich P., Komolmit P., Poovorawan Y. (2013). Prevalence of Aflatoxin Induced p53 Mutation at Codon 249 (R249s) in Hepatocellular Carcinoma Patients with and without Hepatitis B Surface Antigen (HBsAg). Asian Pac. J. Cancer Prev..

[60] Mosbeh A., Abdel-Mageed W., Ezzat S., Kohla M., Sultan M., Abdel-Rahman M. (2023). Low Frequency of Aflatoxin Induced TP53 Gene Codon 249 Mutation in Hepatocellular Carcinoma from Egyptian Patients Living in the Nile Delta Region. Asian Pac. J. Cancer Prev..

[61] Saenen N.D., Martens D.S., Neven K.Y., Alfano R., Bové H., Janssen B.G., Roels H.A., Plusquin M., Vrijens K., Nawrot T.S. (2019). Air pollution-induced placental alterations: An interplay of oxidative stress, epigenetics, and the aging phenotype?. Clin. Epigenet..

[62] Sun Q., Ren X., Sun Z., Duan J. (2021). The critical role of epigenetic mechanism in PM2.5-induced cardiovascular diseases. Genes Environ..

[63] Duan R., Niu H., Dong F., Yu T., Li X., Wu H., Zhang Y., Yang T. (2023). Short-term exposure to fine particulate matter and genome-wide DNA methylation in chronic obstructive pulmonary disease: A panel study conducted in Beijing, China. Front. Public Health.

[64] Li L., Huang L., Lei R., Zhang P., Yang Y., Liu H., Zhang Y. (2024). DEHP and DBP, common phthalates, induce glucose metabolism disorders in rats via oxidative damage of PI3K/Akt/GLUT4 signaling. Environ. Pollut..

[65] Mohammed A., Atkin S.L., Brennan E. (2025). Dysregulation of microRNA (miRNA) Due to Phthalate/Phthalate Metabolite Exposure and Associated Health Effects: A Narrative Review. J. Xenobiotics.

[66] Hlisníková H., Petrovičová I., Kolena B., Šidlovská M., Sirotkin A. (2020). Effects and Mechanisms of Phthalates’ Action on Reproductive Processes and Reproductive Health: A Literature Review. Int. J. Environ. Res. Public Health.

[67] Dworzański W., Cholewińska E., Fotschki B., Juśkiewicz J., Listos P., Ognik K. (2020). Assessment of DNA Methylation and Oxidative Changes in the Heart and Brain of Rats Receiving a High-Fat Diet Supplemented with Various Forms of Chromium. Animals.

[68] Tripathi S., Parmar D., Raval S., Mishra R., Singh G. (2024). Attenuation of chromium (VI) and arsenic (III)-induced oxidative stress and hepatic apoptosis by phloretin, biochanin-A, and coenzyme Q10 via activation of SIRT1/Nrf2/HO-1/NQO1 signaling. J. Biochem. Mol. Toxicol..

[69] Wang Z., Yang C. (2023). Epigenetic and epitranscriptomic mechanisms of chromium carcinogenesis. Adv. Pharmacol..

[70] Hassan S., Thacharodi A., Priya A., Meenatchi R., Hegde T.A., Thangamani R., Nguyen H., Pugazhendhi A. (2024). Endocrine disruptors: Unravelling the link between chemical exposure and Women’s reproductive health. Environ. Res..

[71] Paramasivam A., Murugan R., Jeraud M., Dakkumadugula A., Periyasamy R., Arjunan S. (2024). Additives in Processed Foods as a Potential Source of Endocrine-Disrupting Chemicals: A Review. J. Xenobiotics.

[72] Toporova L., Balaguer P. (2020). Nuclear receptors are the major targets of endocrine disrupting chemicals. Mol. Cell. Endocrinol..

[73] Grimaldi M., Boulahtouf A., Delfosse V., Thouennon E., Bourguet W., Balaguer P. (2015). Reporter Cell Lines for the Characterization of the Interactions between Human Nuclear Receptors and Endocrine Disruptors. Front. Endocrinol..

[74] Wang X., Ha D., Yoshitake R., Chan Y.S., Sadava D., Chen S. (2021). Exploring the Biological Activity and Mechanism of Xenoestrogens and Phytoestrogens in Cancers: Emerging Methods and Concepts. Int. J. Mol. Sci..

[75] Lacouture A., Lafront C., Peillex C., Pelletier M., Audet-Walsh É. (2022). Impacts of endocrine-disrupting chemicals on prostate function and cancer. Environ. Res..

[76] Zhang X., Cheng C., Zhang G., Xiao M., Li L., Wu S., Lu X. (2021). Co-exposure to BPA and DEHP enhances susceptibility of mammary tumors via up-regulating Esr1/HDAC6 pathway in female rats. Ecotoxicol. Environ. Saf..

[77] Betancourt A.M., Eltoum I.A., Desmond R.A., Russo J., Lamartiniere C.A. (2010). In Utero Exposure to Bisphenol A Shifts the Window of Susceptibility for Mammary Carcinogenesis in the Rat. Environ. Health Perspect..

[78] Ji X., Jiang P., Li Y., Su L., Yue H., Sang N. (2023). Maternal Bisphenol B Exposure and Mammary Gland Development of Offspring: A Time-Series Analysis. Environ. Health.

[79] Chuang S.-C., Chen H.-C., Sun C.-W., Chen Y.-A., Wang Y.-H., Chiang C.-J., Chen C.-C., Wang S.-L., Chen C.-J., Hsiung C.A. (2020). Phthalate exposure and prostate cancer in a population-based nested case-control study. Environ. Res..

[80] Yang L., Liu X., Peng Z., Liu Z., Song P., Zhou J., Ma K., Yu Y., Dong Q. (2024). Exposure to di-2-ethylhexyl phthalate (DEHP) increases the risk of cancer. BMC Public Health.

[81] Dutta S., Haggerty D.K., Rappolee D.A., Ruden D.M. (2020). Phthalate Exposure and Long-Term Epigenomic Consequences: A Review. Front. Genet..

[82] Stiefel C., Stintzing F. (2023). Endocrine-active and endocrine-disrupting compounds in food—Occurrence, formation and relevance. NFS J..

[83] Li Y., Ortiz G.G.R., Uyen P.T.M., Cong P.T., Othman S.I., Allam A.A., Unar A., Afridi H.I. (2023). Environmental impact of endocrine-disrupting chemicals and heavy metals in biological samples of petrochemical industry workers with perspective management. Environ. Res..

[84] Tarhonska K., Lesicka M., Janasik B., Roszak J., Reszka E., Braun M., Kołacińska-Wow A., Jabłońska E. (2022). Cadmium and breast cancer—Current state and research gaps in the underlying mechanisms. Toxicol. Lett..

[85] Nandheeswari K., Jayapradha P., Nalla S.V., Dubey I., Kushwaha S. (2024). Arsenic-Induced Thyroid Hormonal Alterations and Their Putative Influence on Ovarian Follicles in Balb/c Mice. Biol. Trace Elem. Res..

[86] Parodi D., Greenfield M., Evans C., Chichura A., Alpaugh A., Williams J., Cyrus K., Martin M. (2017). Alteration of Mammary Gland Development and Gene Expression by In Utero Exposure to Cadmium. Int. J. Mol. Sci..

[87] Hussein M.A., Kamalakkannan A., Valinezhad K., Kannan J., Paleati N., Saad R., Kajdacsy-Balla A., Munirathinam G. (2024). The dynamic face of cadmium-induced Carcinogenesis: Mechanisms, emerging trends, and future directions. Curr. Res. Toxicol..

[88] Adams S.V., Shafer M.M., Bonner M.R., LaCroix A.Z., Manson J.E., Meliker J.R., Neuhouser M.L., Newcomb P.A. (2016). Urinary Cadmium and Risk of Invasive Breast Cancer in the Women’s Health Initiative. Am. J. Epidemiol..

[89] Lu Y., Dang Y., Chen Y., Chen Y., Hui X., Li X., Fan X., Yang J., Ling X., Ma L. (2025). The impact of cadmium exposure on breast cancer risk: Exploring dose-response relationships and mediating effects. Ecotoxicol. Environ. Saf..

[90] Nigam M., Mishra A.P., Deb V.K., Dimri D.B., Tiwari V., Bungau S.G., Bungau A.F., Radu A.-F. (2023). Evaluation of the association of chronic inflammation and cancer: Insights and implications. Biomed. Pharmacother..

[91] Gangwar R.S., Bevan G.H., Palanivel R., Das L., Rajagopalan S. (2020). Oxidative stress pathways of air pollution mediated toxicity: Recent insights. Redox Biol..

[92] Florescu D.N., Boldeanu M.-V., Șerban R.-E., Florescu L.M., Serbanescu M.-S., Ionescu M., Streba L., Constantin C., Vere C.C. (2023). Correlation of the Pro-Inflammatory Cytokines IL-1β, IL-6, and TNF-α, Inflammatory Markers, and Tumor Markers with the Diagnosis and Prognosis of Colorectal Cancer. Life.

[93] Lang T., Lipp A.-M., Wechselberger C. (2025). Xenobiotic Toxicants and Particulate Matter: Effects, Mechanisms, Impacts on Human Health, and Mitigation Strategies. J. Xenobiotics.

[94] Goshtasbi H., Hashemzadeh N., Fathi M., Movafeghi A., Barar J., Omidi Y. (2025). Mitigating oxidative stress toxicities of environmental pollutants by antioxidant nanoformulations. Nano TransMed.

[95] Yu W., Tu Y., Long Z., Liu J., Kong D., Peng J., Wu H., Zheng G., Zhao J., Chen Y. (2022). Reactive Oxygen Species Bridge the Gap between Chronic Inflammation and Tumor Development. Oxid. Med. Cell. Longev..

[96] Yu F., Zhu Y., Li S., Hao L., Li N., Ye F., Jiang Z., Hu X. (2024). Dysfunction and regulatory interplay of T and B cells in chronic hepatitis B: Immunotherapy and emerging antiviral strategies. Front. Cell. Infect. Microbiol..

[97] Rivadeneira D.B., Thosar S., Quann K., Gunn W.G., Dean V.G., Xie B., Parise A., McGovern A.C., Spahr K., Lontos K. (2025). Oxidative-stress-induced telomere instability drives T cell dysfunction in cancer. Immunity.

[98] Liu R., Peng L., Zhou L., Huang Z., Zhou C., Huang C. (2022). Oxidative Stress in Cancer Immunotherapy: Molecular Mechanisms and Potential Applications. Antioxidants.

[99] Chandimali N., Bak S.G., Park E.H., Lim H.-J., Won Y.-S., Kim E.-K., Park S.-I., Lee S.J. (2025). Free radicals and their impact on health and antioxidant defenses: A review. Cell Death Discov..

[100] Wang X., Shen Y., Chen Y., Yang S. (2024). Inflammation-induced cellular changes: Genetic mutations; oncogene impact, and novel glycoprotein biomarkers. Adv. Biomark. Sci. Technol..

[101] Nishida A., Andoh A. (2025). The Role of Inflammation in Cancer: Mechanisms of Tumor Initiation, Progression, and Metastasis. Cells.

[102] Tufail M., Jiang C.-H., Li N. (2025). Immune evasion in cancer: Mechanisms and cutting-edge therapeutic approaches. Signal Transduct. Target. Ther..

[103] Gupta I., Hussein O., Sastry K.S., Bougarn S., Gopinath N., Chin-Smith E., Sinha Y., Korashy H.M., Maccalli C. (2023). Deciphering the complexities of cancer cell immune evasion: Mechanisms and therapeutic implications. Adv. Cancer Biol.-Metastasis.

[104] Madkhali O.A., Moni S.S., Almoshari Y., Sabei F.Y., Safhi A.Y. (2025). Dual role of CXCL10 in cancer progression: Implications for immunotherapy and targeted treatment. Cancer Biol. Ther..

[105] Wang Q., Shao X., Zhang Y., Zhu M., Wang F.X.C., Mu J., Li J., Yao H., Chen K. (2023). Role of tumor microenvironment in cancer progression and therapeutic strategy. Cancer Med..

[106] Lorenc P., Sikorska A., Molenda S., Guzniczak N., Dams-Kozlowska H., Florczak A. (2024). Physiological and tumor-associated angiogenesis: Key factors and therapy targeting VEGF/VEGFR pathway. Biomed. Pharmacother..

[107] Ebrahimi M., Khalili N., Razi S., Keshavarz-Fathi M., Khalili N., Rezaei N. (2020). Effects of lead and cadmium on the immune system and cancer progression. J. Environ. Health Sci. Eng..

[108] Matei E., Râpă M., Mateș I.M., Popescu A.-F., Bădiceanu A., Balint A.I., Covaliu-Mierlă C.I. (2025). Heavy Metals in Particulate Matter—Trends and Impacts on Environment. Molecules.

[109] Potter N.A., Meltzer G.Y., Avenbuan O.N., Raja A., Zelikoff J.T. (2021). Particulate Matter and Associated Metals: A Link with Neurotoxicity and Mental Health. Atmosphere.

[110] Yuan Z., Li Y., Zhang S., Wang X., Dou H., Yu X., Zhang Z., Yang S., Xiao M. (2023). Extracellular matrix remodeling in tumor progression and immune escape: From mechanisms to treatments. Mol. Cancer.

[111] Huang R., Kang T., Chen S. (2024). The role of tumor-associated macrophages in tumor immune evasion. J. Cancer Res. Clin. Oncol..

[112] Jia H., Chen X., Zhang L., Chen M. (2025). Cancer associated fibroblasts in cancer development and therapy. J. Hematol. Oncol..

[113] Wang X., Zhou Y., Wang Y., Yang J., Li Z., Liu F., Wang A., Gao Z., Wu C., Yin H. (2025). Overcoming cancer treatment resistance: Unraveling the role of cancer-associated fibroblasts. J. Natl. Cancer Cent..

[114] Bui B.P., Nguyen P.L., Lee K., Cho J. (2022). Hypoxia-Inducible Factor-1: A Novel Therapeutic Target for the Management of Cancer, Drug Resistance, and Cancer-Related Pain. Cancers.

[115] Gong Q., Shi L., Wen L., Zhang Y., Chen Z., Wei X., Song X., Dong J., Liang C. (2025). Recent advances in metal ions overloading for tumors: Mechanisms, nanomaterials, and therapies. Mater. Today Bio.

[116] Florea A.-M., Büsselberg D. (2008). Arsenic trioxide in environmentally and clinically relevant concentrations interacts with calcium homeostasis and induces cell type specific cell death in tumor and non-tumor cells. Toxicol. Lett..

[117] Dash U.C., Bhol N.K., Swain S.K., Samal R.R., Nayak P.K., Raina V., Panda S.K., Kerry R.G., Duttaroy A.K., Jena A.B. (2025). Oxidative stress and inflammation in the pathogenesis of neurological disorders: Mechanisms and implications. Acta Pharm. Sin. B.

[118] Sule R.O., Rivera G.D.T., Vaidya T., Gartrell E., Gomes A.V. (2025). Environmental Toxins and Oxidative Stress: The Link to Cardiovascular Diseases. Antioxidants.

[119] Omran K., Jiang Y.-J., Ho T.-L., Kousar I., Tang C.-H., Tan M. (2025). Long-Term Particulate Matter (PM) Exposure Promotes Non-Small-Cell Lung Cancer (NSCLC) Angiogenesis Through Up-Regulation of VEGFA. Cancers.

[120] Guo T., Xu J. (2024). Cancer-associated fibroblasts: A versatile mediator in tumor progression, metastasis, and targeted therapy. Cancer Metastasis Rev..

[121] Mafe A.N., Büsselberg D. (2024). Mycotoxins in Food: Cancer Risks and Strategies for Control. Foods.

[122] Han C., Yu T., Qin W., Liao X., Huang J., Liu Z., Yu L., Liu X., Chen Z., Yang C. (2020). Genome-wide association study of the TP53 R249S mutation in hepatocellular carcinoma with aflatoxin B1 exposure and infection with hepatitis B virus. J. Gastrointest. Oncol..

[123] Zhou X., Speer R.M., Volk L., Hudson L.G., Liu K.J. (2021). Arsenic co-carcinogenesis: Inhibition of DNA repair and interaction with zinc finger proteins. Semin. Cancer Biol..

[124] Ahuja V., Singh A., Paul D., Dasgupta D., Urajová P., Ghosh S., Singh R., Sahoo G., Ewe D., Saurav K. (2023). Recent Advances in the Detection of Food Toxins Using Mass Spectrometry. Chem. Res. Toxicol..

[125] Vineis P., Robinson O., Chadeau-Hyam M., Dehghan A., Mudway I., Dagnino S. (2020). What is new in the exposome?. Environ. Int..

[126] Kobets T., Smith B.P.C., Williams G.M. (2022). Food-Borne Chemical Carcinogens and the Evidence for Human Cancer Risk. Foods.

[127] Zhou S., Zhu Q., Liu H., Jiang S., Zhang X., Peng C., Yang G., Li J., Cheng L., Zhong R. (2021). Associations of polycyclic aromatic hydrocarbons exposure and its interaction with XRCC1 genetic polymorphism with lung cancer: A case-control study. Environ. Pollut..

[128] Montano L., Baldini G.M., Piscopo M., Liguori G., Lombardi R., Ricciardi M., Esposito G., Pinto G., Fontanarosa C., Spinelli M. (2025). Polycyclic Aromatic Hydrocarbons (PAHs) in the Environment: Occupational Exposure, Health Risks and Fertility Implications. Toxics.

[129] Ghoreyshi N., Heidari R., Farhadi A., Chamanara M., Farahani N., Vahidi M., Behroozi J. (2025). Next-generation sequencing in cancer diagnosis and treatment: Clinical applications and future directions. Discov. Oncol..

[130] Yaacov A., Cohen G.B., Landau J., Hope T., Simon I., Rosenberg S. (2024). Cancer mutational signatures identification in clinical assays using neural embedding-based representations. Cell Rep. Med..

[131] Snyder R. (2012). Leukemia and Benzene. Int. J. Environ. Res. Public Health.

[132] Cordiano R., Papa V., Cicero N., Spatari G., Allegra A., Gangemi S. (2022). Effects of Benzene: Hematological and Hypersensitivity Manifestations in Resident Living in Oil Refinery Areas. Toxics.

[133] Sun R., Xu K., Ji S., Pu Y., Yu L., Yin L., Zhang J., Pu Y. (2021). Toxicity in hematopoietic stem cells from bone marrow and peripheral blood in mice after benzene exposure: Single-cell transcriptome sequencing analysis. Ecotoxicol. Environ. Saf..

[134] Abrehame S., Manoj V.R., Hailu M., Chen Y.-Y., Lin Y.-C., Chen Y.-P. (2023). Aflatoxins: Source, Detection, Clinical Features and Prevention. Processes.

[135] Benkerroum N. (2020). Chronic and Acute Toxicities of Aflatoxins: Mechanisms of Action. Int. J. Environ. Res. Public Health.

[136] Weng M.-W., Lee H.-W., Choi B., Wang H.-T., Hu Y., Mehta M., Desai D., Amin S., Zheng Y., Tang M.-S. (2017). AFB1 hepatocarcinogenesis is via lipid peroxidation that inhibits DNA repair, sensitizes mutation susceptibility and induces aldehyde-DNA adducts at p53 mutational hotspot codon 249. Oncotarget.

[137] Mlangeni A.T. (2023). Methylation of arsenic in rice: Mechanisms, factors, and mitigation strategies. Toxicol. Rep..

[138] Dai J., Chen C., Zhai Z.-Q., Gao A.-X., Johnson D.R., Kopittke P.M., Zhao F.-J., Wang P. (2025). The balance between microbial arsenic methylation and demethylation in paddy soils underpins global arsenic risk and straighthead disease in rice. Proc. Natl. Acad. Sci. USA.

[139] Kazemifar A.M., Shafikhani A.A., Mozhdehipanah H., Khamesi S., Arami M. (2020). Evaluation of different types of arsenic methylation and its relationship with metabolic syndrome in an area chronically exposed to arsenic. Environ. Anal. Health Toxicol..

[140] da Costa J.M., Kato L.S., Galvan D., Lelis C.A., Saraiva T., Conte-Junior C.A. (2023). Occurrence of phthalates in different food matrices: A systematic review of the main sources of contamination and potential risks. Compr. Rev. Food Sci. Food Saf..

[141] Koniecki D., Wang R., Moody R.P., Zhu J. (2011). Phthalates in cosmetic and personal care products: Concentrations and possible dermal exposure. Environ. Res..

[142] Yuan Q., Chen Y., Li X., Zhang Z., Chu H. (2019). Ambient fine particulate matter (PM2.5) induces oxidative stress and pro-inflammatory response via up-regulating the expression of CYP1A1/1B1 in human bronchial epithelial cells in vitro. Mutat. Res. Toxicol. Environ. Mutagen..

[143] Xu Z., Ding W., Deng X. (2019). PM_2.5_, Fine Particulate Matter: A Novel Player in the Epithelial-Mesenchymal Transition?. Front. Physiol..

[144] Zhai X., Wang J., Sun J., Xin L. (2022). PM_2.5_ induces inflammatory responses via oxidative stress-mediated mitophagy in human bronchial epithelial cells. Toxicol. Res..

[145] Smith M., Love D.C., Rochman C.M., Neff R.A. (2018). Microplastics in Seafood and the Implications for Human Health. Curr. Environ. Health Rep..

[146] Haleem N., Kumar P., Zhang C., Jamal Y., Hua G., Yao B., Yang X. (2024). Microplastics and associated chemicals in drinking water: A review of their ocurrence and human health implications. Sci. Total Environ..

[147] Pironti C., Ricciardi M., Motta O., Miele Y., Proto A., Montano L. (2021). Microplastics in the Environment: Intake through the Food Web, Human Exposure and Toxicological Effects. Toxics.

[148] Sun H., Brocato J., Costa M. (2015). Oral Chromium Exposure and Toxicity. Curr. Environ. Health Rep..

[149] Yatera K., Morimoto Y., Ueno S., Noguchi S., Kawaguchi T., Tanaka F., Suzuki H., Higashi T. (2018). Cancer Risks of Hexavalent Chromium in the Respiratory Tract. J. UOEH.

[150] Krawic C., Zhitkovich A. (2023). Chemical mechanisms of DNA damage by carcinogenic chromium(VI). Adv. Pharmacol..

[151] Colopi A., Guida E., Cacciotti S., Fuda S., Lampitto M., Onorato A., Zucchi A., Balistreri C.R., Grimaldi P., Barchi M. (2024). Dietary Exposure to Pesticide and Veterinary Drug Residues and Their Effects on Human Fertility and Embryo Development: A Global Overview. Int. J. Mol. Sci..

[152] Hongsibsong S., Sittitoon N., Sapbamrer R. (2017). Association of health symptoms with low-level exposure to organophosphates, DNA damage, AChE activity, and occupational knowledge and practice among rice, corn, and double-crop farmers. J. Occup. Health.

[153] Yang K.J., Lee J., Park H.L. (2020). Organophosphate Pesticide Exposure and Breast Cancer Risk: A Rapid Review of Human, Animal, and Cell-Based Studies. Int. J. Environ. Res. Public Health.

[154] Mangiaterra S., Boffetta P., Seyyedsalehi M.S. (2025). Occupational benzene exposure and risk of nervous system cancers: A systematic review and meta-analysis. Cancer Epidemiol..

[155] Patel A.B., Shaikh S., Jain K.R., Desai C., Madamwar D. (2020). Polycyclic Aromatic Hydrocarbons: Sources, Toxicity, and Remediation Approaches. Front. Microbiol..

[156] Aslam R., Sharif F., Baqar M., Shahzad L. (2022). Source identification and risk assessment of polycyclic aromatic hydrocarbons (PAHs) in air and dust samples of Lahore City. Sci. Rep..

[157] Mathialagan R.D., Hamid Z.A., Ng Q.M., Rajab N.F., Shuib S., Razak S.R.B.A. (2020). Bone Marrow Oxidative Stress and Acquired Lineage-Specific Genotoxicity in Hematopoietic Stem/Progenitor Cells Exposed to 1,4-Benzoquinone. Int. J. Environ. Res. Public Health.

[158] Wu W., Zhang L., Zhang M., Zhu L.-J., Xia H.-L., Lou J.-L., Liu J.-R., Xiao Y. (2016). Proteome Changes of Human Bone Marrow Mesenchymal Stem Cells Induced by 1,4-Benzoquinone. BioMed Res. Int..

[159] Dewi R., Hamid Z.A., Rajab N., Shuib S., Razak S.A. (2020). Genetic, epigenetic, and lineage-directed mechanisms in benzene-induced malignancies and hematotoxicity targeting hematopoietic stem cells niche. Hum. Exp. Toxicol..

[160] Godono A., Dito A., Martini G., Picciaiola M.V., Di Lorenzo A., Ciocan C., Boffetta P., Seyyedsalehi M.S. (2024). Occupational benzene exposure and risk of head and neck cancer: A systematic review and meta-analysis. Environ. Res..

[161] Sun R., Xu K., Ji S., Pu Y., Man Z., Ji J., Chen M., Yin L., Zhang J., Pu Y. (2020). Benzene exposure induces gut microbiota dysbiosis and metabolic disorder in mice. Sci. Total Environ..

[162] Mafe A.N., Büsselberg D. (2025). The Effect of Microbiome-Derived Metabolites in Inflammation-Related Cancer Prevention and Treatment. Biomolecules.

[163] Gemede H.F. (2025). Toxicity, Mitigation, and Chemical Analysis of Aflatoxins and Other Toxic Metabolites Produced by Aspergillus: A Comprehensive Review. Toxins.

[164] Jedua H., Mafe A.N., Edo G.I., Ali A.B.M., Iwanegbe I., Akpoghelie P.O., Owheruo J.O., Onukwubiri M., Yousif E., Igbuku U.A. (2026). Microbial contamination pathways: Assessing the impact of building-damaging microorganisms on food safety and structural integrity in humid home environments. J. Stored Prod. Res..

[165] Jallow A., Xie H., Tang X., Qi Z., Li P. (2021). Worldwide aflatoxin contamination of agricultural products and foods: From occurrence to control. Compr. Rev. Food Sci. Food Saf..

[166] Gramantieri L., Gnudi F., Vasuri F., Mandrioli D., Fornari F., Tovoli F., Suzzi F., Vornoli A., D’Errico A., Piscaglia F. (2022). Aflatoxin B1 DNA-Adducts in Hepatocellular Carcinoma from a Low Exposure Area. Nutrients.

[167] Aguilar F., Hussain S.P., Cerutti P. (1993). Aflatoxin B1 induces the transversion of G→T in codon 249 of the p53 tumor suppressor gene in human hepatocytes. Proc. Natl. Acad. Sci. USA.

[168] Moloi T.P., Ziqubu K., Mazibuko-Mbeje S.E., Mabaso N.H., Ndlovu Z. (2024). Aflatoxin B1-induced hepatotoxicity through mitochondrial dysfunction, oxidative stress, and inflammation as central pathological mechanisms: A review of experimental evidence. Toxicology.

[169] Hua Z., Liu R., Chen Y., Liu G., Li C., Song Y., Cao Z., Li W., Li W., Lu C. (2021). Contamination of Aflatoxins Induces Severe Hepatotoxicity Through Multiple Mechanisms. Front. Pharmacol..

[170] Kuniholm M.H., Lesi O.A., Mendy M., Akano A.O., Sam O., Hall A.J., Whittle H., Bah E., Goedert J.J., Hainaut P. (2008). Aflatoxin Exposure and Viral Hepatitis in the Etiology of Liver Cirrhosis in The Gambia, West Africa. Environ. Health Perspect..

[171] Foerster C., Koshiol J., Guerrero A.R., Kogan M.J., Ferreccio C. (2016). The case for aflatoxins in the causal chain of gallbladder cancer. Med. Hypotheses.

[172] Shaji E., Santosh M., Sarath K.V., Prakash P., Deepchand V., Divya B.V. (2021). Arsenic contamination of groundwater: A global synopsis with focus on the Indian Peninsula. Geosci. Front..

[173] Oladimeji T.E., Oyedemi M., Emetere M.E., Agboola O., Adeoye J.B., Odunlami O.A. (2024). Review on the impact of heavy metals from industrial wastewater effluent and removal technologies. Heliyon.

[174] Martinez V.D., Lam W.L. (2021). Health Effects Associated With Pre- and Perinatal Exposure to Arsenic. Front. Genet..

[175] Hu Y., Li J., Lou B., Wu R., Wang G., Lu C., Wang H., Pi J., Xu Y. (2020). The Role of Reactive Oxygen Species in Arsenic Toxicity. Biomolecules.

[176] Islam R., Zhao L., Wang Y., Lu-Yao G., Liu L.-Z. (2022). Epigenetic Dysregulations in Arsenic-Induced Carcinogenesis. Cancers.

[177] Mafe A.N., Makinde O., Adeleke R.A. (2025). Geophagia among pregnant women: Evaluating the microbiological and toxicological safety of calabash chalk and its implications on maternal health. Environ. Geochem. Health.

[178] Khan K.M., Chakraborty R., Bundschuh J., Bhattacharya P., Parvez F. (2020). Health effects of arsenic exposure in Latin America: An overview of the past eight years of research. Sci. Total Environ..

[179] Khaleda L., Alam M.M., Tasnim Z., Ezaj M.M.A., Apu M.A.R., Akter R., Bakar M.A., Alam M.J., Chowdhury R.H., Datta A. (2025). Detrimental effects of chronic arsenic exposure through daily diet on hepatic and renal health: An animal model study. Toxicol. Rep..

[180] Wang Y., Qian H. (2021). Phthalates and Their Impacts on Human Health. Healthcare.

[181] Ali N., Alhakamy N.A., Ismail I.M.I., Nazar E., Summan A.S., Eqani S.A.M.A.S., Malarvannan G. (2021). Exposure to Phthalate and Organophosphate Esters via Indoor Dust and PM10 Is a Cause of Concern for the Exposed Saudi Population. Int. J. Environ. Res. Public Health.

[182] Pagoni A., Arvaniti O.S., Kalantzi O.-I. (2022). Exposure to phthalates from personal care products: Urinary levels and predictors of exposure. Environ. Res..

[183] Arrigo F., Impellitteri F., Piccione G., Faggio C. (2023). Phthalates and their effects on human health: Focus on erythrocytes and the reproductive system. Comp. Biochem. Physiol. Part C Toxicol. Pharmacol..

[184] Martínez-Ibarra A., Martínez-Razo L.D., MacDonald-Ramos K., Morales-Pacheco M., Vázquez-Martínez E.R., López-López M., Dorantes M.R., Cerbón M. (2021). Multisystemic alterations in humans induced by bisphenol A and phthalates: Experimental, epidemiological and clinical studies reveal the need to change health policies. Environ. Pollut..

[185] Zheng W.-R., Li Y.-Z., Xu J., Liu K.-X., Liu F.-H., Xing W.-Y., Liu J.-X., Wu L., Li X.-Y., Huang D.-H. (2025). Urinary concentrations of phthalate metabolites and the survival of high-grade serous ovarian cancer with advanced stage. Environ. Pollut..

[186] Wang M., Kim R.Y., Kohonen-Corish M.R.J., Chen H., Donovan C., Oliver B.G. (2025). Particulate matter air pollution as a cause of lung cancer: Epidemiological and experimental evidence. Br. J. Cancer.

[187] Soares M., Oliveira H., Alves C. (2025). Airborne particulate matter inhalation bioaccessibility: A review of methodological aspects. Chem. Biol. Interact..

[188] Sangkham S., Phairuang W., Sherchan S.P., Pansakun N., Munkong N., Sarndhong K., Islam M.A., Sakunkoo P. (2024). An update on adverse health effects from exposure to PM_2.5_. Environ. Adv..

[189] González-Ruíz J., Baccarelli A.A., Cantu-de-Leon D., Prada D. (2023). Air Pollution and Lung Cancer: Contributions of Extracellular Vesicles as Pathogenic Mechanisms and Clinical Utility. Curr. Environ. Health Rep..

[190] Li R., Zhao C., Zhang Y., Huang W., Wang J., Cao G., Cai Z. (2024). PM_2.5_-induced DNA oxidative stress in A549 cells and regulating mechanisms by GST DNA methylation and Keap1/Nrf2 pathway. Toxicol. Mech. Methods.

[191] Gavito-Covarrubias D., Ramírez-Díaz I., Guzmán-Linares J., Limón I.D., Manuel-Sánchez D.M., Molina-Herrera A., Coral-García M.Á., Anastasio E., Anaya-Hernández A., López-Salazar P. (2024). Epigenetic mechanisms of particulate matter exposure: Air pollution and hazards on human health. Front. Genet..

[192] Cui C., Yang R., Chen H., Li D., Sun X., Wang Y., Pan Q. (2025). Air toxins disorder the NF-kB Pathway leads to immune disorders and immune diseases in the human health. Ecotoxicol. Environ. Saf..

[193] Chen C.-Y., Huang K.-Y., Chen C.-C., Chang Y.-H., Li H.-J., Wang T.-H., Yang P.-C. (2024). The role of PM2.5 exposure in lung cancer: Mechanisms, genetic factors, and clinical implications. EMBO Mol. Med..

[194] Shankar A., Dubey A., Saini D., Singh M., Prasad C.P., Roy S., Bharati S.J., Rinki M., Singh N., Seth T. (2019). Environmental and occupational determinants of lung cancer. Transl. Lung Cancer Res..

[195] Mukherjee S., Dasgupta S., Mishra P.K., Chaudhury K. (2021). Air pollution-induced epigenetic changes: Disease development and a possible link with hypersensitivity pneumonitis. Environ. Sci. Pollut. Res..

[196] Bora S.S., Gogoi R., Sharma M.R., Anshu, Borah M.P., Deka P., Bora J., Naorem R.S., Das J., Teli A.B. (2024). Microplastics and human health: Unveiling the gut microbiome disruption and chronic disease risks. Front. Cell. Infect. Microbiol..

[197] Zuri G., Karanasiou A., Lacorte S. (2023). Microplastics: Human exposure assessment through air, water, and food. Environ. Int..

[198] Tang K.H.D. (2025). Counteracting the Harms of Microplastics on Humans: An Overview from the Perspective of Exposure. Microplastics.

[199] Xu J., Qu J., Jin H., Mao W. (2025). Associations between microplastics in human feces and colorectal cancer risk. J. Hazard. Mater..

[200] Mafe A.N., Büsselberg D. (2025). Food Preservatives and the Rising Tide of Early-Onset Colorectal Cancer: Mechanisms, Controversies, and Emerging Innovations. Foods.

[201] Jomova K., Alomar S.Y., Nepovimova E., Kuca K., Valko M. (2025). Heavy metals: Toxicity and human health effects. Arch. Toxicol..

[202] Angon P.B., Islam M.S., KC S., Das A., Anjum N., Poudel A., Suchi S.A. (2024). Sources, effects and present perspectives of heavy metals contamination: Soil, plants and human food chain. Heliyon.

[203] Alengebawy A., Abdelkhalek S.T., Qureshi S.R., Wang M.-Q. (2021). Heavy Metals and Pesticides Toxicity in Agricultural Soil and Plants: Ecological Risks and Human Health Implications. Toxics.

[204] Miletić A., Lučić M., Onjia A. (2023). Exposure Factors in Health Risk Assessment of Heavy Metal(loid)s in Soil and Sediment. Metals.

[205] Tchounwou P.B., Yedjou C.G., Patlolla A.K., Sutton D.J. (2012). Heavy Metal Toxicity and the Environment. Mol. Clin. Environ. Toxicol..

[206] Florea A.-M., Büsselberg D. (2006). Occurrence, use and potential toxic effects of metals and metal compounds. BioMetals.

[207] Haidar Z., Fatema K., Shoily S.S., Sajib A.A. (2023). Disease-associated metabolic pathways affected by heavy metals and metalloid. Toxicol. Rep..

[208] Mutethya E., Liu Q., Yongo E., Guo Z., Yu H., Zhang Y., Lu Z., Ye C. (2025). Ecological risk assessment of heavy metal contamination in Xiaohai Lagoon, Hainan island, China. Front. Mar. Sci..

[209] Yu L., Liu J., Yan Y., Burwell A., Castro L., Shi M., Dixon D. (2021). “Metalloestrogenic” effects of cadmium downstream of G protein-coupled estrogen receptor and mitogen-activated protein kinase pathways in human uterine fibroid cells. Arch. Toxicol..

[210] Tam L.M., Price N.E., Wang Y. (2020). Molecular Mechanisms of Arsenic-Induced Disruption of DNA Repair. Chem. Res. Toxicol..

[211] Yadav R.S., Kushawaha B., Dhariya R., Swain D.K., Yadav B., Anand M., Kumari P., Rai P.K., Singh D., Yadav S. (2024). Lead and calcium crosstalk tempted acrosome damage and hyperpolarization of spermatozoa: Signaling and ultra-structural evidences. Biol. Res..

[212] Mezencev R., Gibbons C. (2023). Interactions between chromium species and DNA in vitro and their potential role in the toxicity of hexavalent chromium. Metallomics.

[213] Coradduzza D., Congiargiu A., Azara E., Mammani I.M.A., De Miglio M.R., Zinellu A., Carru C., Medici S. (2024). Heavy metals in biological samples of cancer patients: A systematic literature review. BioMetals.

[214] Genchi G., Sinicropi M.S., Lauria G., Carocci A., Catalano A. (2020). The Effects of Cadmium Toxicity. Int. J. Environ. Res. Public Health.

[215] Ahn J., Park M.Y., Kang M.-Y., Shin I.-S., An S., Kim H.-R. (2020). Occupational Lead Exposure and Brain Tumors: Systematic Review and Meta-Analysis. Int. J. Environ. Res. Public Health.

[216] Zhou W., Li M., Achal V. (2025). A comprehensive review on environmental and human health impacts of chemical pesticide usage. Emerg. Contam..

[217] Shekhar C., Khosya R., Thakur K., Mahajan D., Kumar R., Kumar S., Sharma A.K. (2024). A systematic review of pesticide exposure, associated risks, and long-term human health impacts. Toxicol. Rep..

[218] Cavalier H., Trasande L., Porta M. (2023). Exposures to pesticides and risk of cancer: Evaluation of recent epidemiological evidence in humans and paths forward. Int. J. Cancer.

[219] Tudi M., Li H., Li H., Wang L., Lyu J., Yang L., Tong S., Yu Q.J., Ruan H.D., Atabila A. (2022). Exposure Routes and Health Risks Associated with Pesticide Application. Toxics.

[220] Lari S., Jonnalagadda P.R., Yamagani P., Medithi S., Vanka J., Pandiyan A., Naidu M., Jee B. (2022). Assessment of dermal exposure to pesticides among farmers using dosimeter and hand washing methods. Front. Public Health.

[221] Sule R.O., Condon L., Gomes A.V. (2022). A Common Feature of Pesticides: Oxidative Stress—The Role of Oxidative Stress in Pesticide-Induced Toxicity. Oxid. Med. Cell. Longev..

[222] Muñoz-Bautista J.M., Bernal-Mercado A.T., Martínez-Cruz O., Burgos-Hernández A., López-Zavala A.A., Ruiz-Cruz S., Ornelas-Paz J.d.J., Borboa-Flores J., Ramos-Enríquez J.R., Del-Toro-Sánchez C.L. (2025). Environmental and Health Impacts of Pesticides and Nanotechnology as an Alternative in Agriculture. Agronomy.

[223] Singh D.D. (2024). Epigenetic Mechanisms of Endocrine-Disrupting Chemicals in Breast Cancer and Their Impact on Dietary Intake. J. Xenobiotics.

[224] Kaur K., Kaur R. (2018). Occupational pesticide exposure, impaired DNA repair, and diseases. Indian J. Occup. Environ. Med..

[225] de Almeida W., Matei J.C., Kitamura R.S.A., Gomes M.P., Leme D.M., de Assis H.C.S., Vicari T., Cestari M.M. (2023). Alkylphenols cause cytotoxicity and genotoxicity induced by oxidative stress in RTG-2 cell line. Chemosphere.

[226] Sadida H.Q., Abdulla A., Al Marzooqi S., Hashem S., Macha M.A., Akil A.S.A.-S., Bhat A.A. (2024). Epigenetic modifications: Key players in cancer heterogeneity and drug resistance. Transl. Oncol..

[227] Pathak A., Tomar S., Pathak S. (2023). Epigenetics and Cancer: A Comprehensive Review. Asian Pac. J. Cancer Biol..

[228] Chen W.-L., Lin G.-L., Lin Y.-J., Su T.-Y., Wang C.-C., Wu W.-T. (2023). Cancer risks in a population-based study of agricultural workers: Results from the Taiwan’s Farmers and Health Cohort study. Scand. J. Work. Environ. Health.

[229] Pedroso T.M.A., Benvindo-Souza M., de Araújo Nascimento F., Woch J., dos Reis F.G., de Melo e Silva D. (2022). Cancer and occupational exposure to pesticides: A bibliometric study of the past 10 years. Environ. Sci. Pollut. Res..

[230] Cheng Y., Zhao Y., Chen C., Zhang F. (2025). Heavy Metals Toxicity: Mechanism, Health Effects, and Therapeutic Interventions. MedComm.

[231] Ringler C., Agbonlahor M., Barron J., Baye K., Meenakshi J.V., Mekonnen D.K., Uhlenbrook S. (2022). The role of water in transforming food systems. Glob. Food Sec..

[232] Rodríguez-Carrillo A., Mustieles V., Salamanca-Fernández E., Olivas-Martínez A., Suárez B., Bajard L., Baken K., Blaha L., Bonefeld-Jørgensen E.C., Couderq S. (2023). Implementation of effect biomarkers in human biomonitoring studies: A systematic approach synergizing toxicological and epidemiological knowledge. Int. J. Hyg. Environ. Health.

[233] Hartwig A., Arand M., Epe B., Guth S., Jahnke G., Lampen A., Martus H.-J., Monien B., Rietjens I.M.C.M., Schmitz-Spanke S. (2020). Mode of action-based risk assessment of genotoxic carcinogens. Arch. Toxicol..

[234] Reis J., Spencer P.S. (2019). Decision-making under uncertainty in environmental health policy: New approaches. Environ. Health Prev. Med..

[235] Jomova K., Makova M., Alomar S.Y., Alwasel S.H., Nepovimova E., Kuca K., Rhodes C.J., Valko M. (2022). Essential metals in health and disease. Chem. Biol. Interact..

[236] Afzal A., Mahreen N. (2024). Emerging insights into the impacts of heavy metals exposure on health, reproductive and productive performance of livestock. Front. Pharmacol..

[237] Veltman C.H.J., Pennings J.L.A., van de Water B., Luijten M. (2023). An Adverse Outcome Pathway Network for Chemically Induced Oxidative Stress Leading to (Non)genotoxic Carcinogenesis. Chem. Res. Toxicol..

[238] Kasperczyk E., Tarhonska K., Jablonska E. (2025). Genotoxicity Induced by Carcinogenic Agents or Occupational Exposure with Sufficient Evidence for Bladder Cancer. J. Clin. Med..

[239] Foreman A.L., Warth B., Hessel E.V.S., Price E.J., Schymanski E.L., Cantelli G., Parkinson H., Hecht H., Klánová J., Vlaanderen J. (2024). Adopting Mechanistic Molecular Biology Approaches in Exposome Research for Causal Understanding. Environ. Sci. Technol..

[240] Newell M.E., Aravindan A., Babbrah A., Halden R.U. (2025). Epigenetic Biomarkers Driven by Environmental Toxins Associated with Alzheimer’s Disease, Parkinson’s Disease, and Amyotrophic Lateral Sclerosis in the United States: A Systematic Review. Toxics.

[241] Deepika D., Bharti K., Sharma S., Kumar S., Pathak R.K., Brull J.B., Sabuz O., Vilana S.G., Kumar V. (2025). Advancing human health risk assessment: The role of new approach methodologies. Front. Toxicol..

[242] Varshavsky J.R., Rayasam S.D.G., Sass J.B., Axelrad D.A., Cranor C.F., Hattis D., Hauser R., Koman P.D., Marquez E.C., Morello-Frosch R. (2023). Current practice and recommendations for advancing how human variability and susceptibility are considered in chemical risk assessment. Environ. Health.

[243] Khoshakhlagh A.H., Mohammadzadeh M., Gruszecka-Kosowska A. (2024). The preventive and carcinogenic effect of metals on cancer: A systematic review. BMC Public Health.

[244] Coleraus F., de Marchi Sanches Azevedo C., Pavlak J.L., Marek C.B., Guimarães A.T.B. (2025). Multigenerational exposure to trace concentrations of DDT residues in Wistar rats: Effects on biometric development and biochemical parameters. Toxicol. Rep..

[245] Sánchez-Alarcón J., Milić M., Kašuba V., Tenorio-Arvide M., Montiel-González J., Bonassi S., Valencia-Quintana R. (2021). A Systematic Review of Studies on Genotoxicity and Related Biomarkers in Populations Exposed to Pesticides in Mexico. Toxics.

[246] Dodos K., Kalamara T.-V., Papalexis P., Keramydas D.A., Papageorgiou E.G., Georgakopoulou V.E. (2025). Asbestos Exposure and Leukemia Incidence: A Systematic Review and Meta-analysis. In Vivo.

[247] Yuan M., Yang B., Rothschild G., Mann J.J., Sanford L.D., Tang X., Huang C., Wang C., Zhang W. (2023). Epigenetic regulation in major depression and other stress-related disorders: Molecular mechanisms, clinical relevance and therapeutic potential. Signal Transduct. Target. Ther..

[248] Tran N.Q.V., Miyake K. (2017). Neurodevelopmental Disorders and Environmental Toxicants: Epigenetics as an Underlying Mechanism. Int. J. Genom..

[249] Miya T.V., Marima R., Marutha T., Luvhengo T.E., Mkhize-Kwitshana Z., Chauke-Malinga N., Mazibuko G., Dlamini Z. (2025). Traditional medicine, environmental exposures, and cultural practices in cancer risk: Insights from low- and middle-income countries. Front. Oncol..

[250] Lucchesi C.A., Vasilatis D.M., Mantrala S., Chandrasekar T., Mudryj M., Ghosh P.M. (2023). Pesticides and Bladder Cancer: Mechanisms Leading to Anti-Cancer Drug Chemoresistance and New Chemosensitization Strategies. Int. J. Mol. Sci..

[251] Martin O., Scholze M., Ermler S., McPhie J., Bopp S.K., Kienzler A., Parissis N., Kortenkamp A. (2021). Ten years of research on synergisms and antagonisms in chemical mixtures: A systematic review and quantitative reappraisal of mixture studies. Environ. Int..

[252] Ashraf W., Abdul R., Ahmad M.-D., Rabbani M., Aamir K., Wang J.-S. (2024). Assessing the Risk and Consequences of Naturally OcCurr.ing Aflatoxins on Liver and Kidney Health in Children: A Cross-sectional Analysis in Lahore Pakistan. Public Health Toxicol..

[253] Poirier M.C. (2012). Chemical-induced DNA damage and human cancer risk. Discov. Med..

[254] Schrenk D. (2018). What is the meaning of ‘A compound is carcinogenic’?. Toxicol. Rep..

[255] Mace K. (1997). Aflatoxin B1-induced DNA adduct formation and p53 mutations in CYP450- expressing human liver cell lines. Carcinogenesis.

[256] Matés J.M., Segura J.A., Alonso F.J., Márquez J. (2010). Roles of dioxins and heavy metals in cancer and neurological diseases using ROS-mediated mechanisms. Free Radic. Biol. Med..

[257] Gupta P., Thompson B.L., Wahlang B., Jordan C.T., Hilt J.Z., Hennig B., Dziubla T. (2018). The environmental pollutant, polychlorinated biphenyls, and cardiovascular disease: A potential target for antioxidant nanotherapeutics. Drug Deliv. Transl. Res..

[258] Zhang S., Xiao X., Yi Y., Wang X., Zhu L., Shen Y., Lin D., Wu C. (2024). Tumor initiation and early tumorigenesis: Molecular mechanisms and interventional targets. Signal Transduct. Target. Ther..

[259] Briffa J., Sinagra E., Blundell R. (2020). Heavy metal pollution in the environment and their toxicological effects on humans. Heliyon.

[260] Asiminicesei D.-M., Fertu D.I., Gavrilescu M. (2024). Impact of Heavy Metal Pollution in the Environment on the Metabolic Profile of Medicinal Plants and Their Therapeutic Potential. Plants.

[261] Cruz J.C., Rocha B.A., Souza M.C.O., Kannan K., Júnior F.B. (2025). Co-exposure to multiple endocrine-disrupting chemicals and oxidative stress: Epidemiological evidence of nonmonotonic dose response curves. Sci. Total Environ..

[262] Shi Z., Li Z., Yang F. (2025). Investigating the potential causal link between BPA and ovarian carcinogenesis: A network toxicology and mendelian randomization study on the CTRC/PRDX1/SKP1 pathway. J. Ovarian Res..

[263] Gengatharan A., Mohamad N.V., Zahari C.N.M.C., Vijayakumar R. (2025). Seaweeds as emerging functional foods and therapeutics for colorectal cancer management. Discov. Food.

[264] Cheong A., Nagel Z.D. (2022). Human Variation in DNA Repair, Immune Function, and Cancer Risk. Front. Immunol..

[265] Mokhosoev I.M., Astakhov D.V., Terentiev A.A., Moldogazieva N.T. (2024). Human Cytochrome P450 Cancer-Related Metabolic Activities and Gene Polymorphisms: A Review. Cells.

[266] Zhang F., Wu X., Niu J., Kang X., Cheng L., Lv Y., Wu M. (2017). GSTM1 polymorphism is related to risks of nasopharyngeal cancer and laryngeal cancer: A meta-analysis. OncoTargets Ther..

[267] Avirmed S., Khuanbai Y., Sanjaajamts A., Selenge B., Dagvadorj B.-U., Ohashi M. (2021). Modifying Effect of Smoking on GSTM1 and NAT2 in Relation to the Risk of Bladder Cancer in Mongolian Population: A Case-Control Study. Asian Pac. J. Cancer Prev..

[268] Li Y., Hecht S.S. (2022). Metabolic Activation and DNA Interactions of Carcinogenic N-Nitrosamines to Which Humans Are Commonly Exposed. Int. J. Mol. Sci..

[269] Li S., Xue F., Zheng Y., Yang P., Lin S., Deng Y., Xu P., Zhou L., Hao Q., Zhai Z. (2019). GSTM1 and GSTT1 null genotype increase the risk of hepatocellular carcinoma: Evidence based on 46 studies. Cancer Cell Int..

[270] Khosravi M.H., Sharafi H., Alavian S.M. (2021). Association of GSTM1 and GSTT1 Null Deletions and GSTP1 rs1695 Polymorphism with the Risk of Hepatocellular Carcinoma: A Systematic Review and Meta-analysis. Hepat. Mon..

[271] Rahbar M.H., Samms-Vaughan M., Kim S., Saroukhani S., Bressler J., Hessabi M., Grove M.L., Shakspeare-Pellington S., Loveland K.A. (2022). Detoxification Role of Metabolic Glutathione S-Transferase (GST) Genes in Blood Lead Concentrations of Jamaican Children with and without Autism Spectrum Disorder. Genes.

[272] Le T.H. (2021). GSTM1 Gene, Diet, and Kidney Disease: Implication for Precision Medicine?: Recent Advances in Hypertension. Hypertension.

[273] Filip C.I., Cătană A., Pîrlog L.-M., Pătrășcanu A.-A., Militaru M.S., Iordănescu I., Dindelegan G.C. (2025). Associations Between DNA Repair Gene Polymorphisms and Breast Cancer Histopathological Subtypes: A Preliminary Study. J. Clin. Med..

[274] Charles M.R., Raza S.T., Pratap P., Eba A. (2024). Association of Genetic Polymorphisms in Base excision Repair Pathways and Cervical Cancer Risk Factors in a Tertiary Care Centre. Asian Pac. J. Cancer Biol..

[275] Liu Q., Peng Q., Zhang B., Tan Y. (2023). X-ray cross-complementing family: The bridge linking DNA damage repair and cancer. J. Transl. Med..

[276] Larsen K., Rydz E., Peters C.E. (2023). Inequalities in Environmental Cancer Risk and Carcinogen Exposures: A Scoping Review. Int. J. Environ. Res. Public Health.

[277] Omotoso O., Teibo J.O., Atiba F.A., Oladimeji T., Paimo O.K., Ataya F.S., Batiha G.E.-S., Alexiou A. (2023). Addressing cancer care inequities in sub-Saharan Africa: Current challenges and proposed solutions. Int. J. Equity Health.

[278] Ericson B., Hu H., Nash E., Ferraro G., Sinitsky J., Taylor M.P. (2021). Blood lead levels in low-income and middle-income countries: A systematic review. Lancet Planet. Health.

[279] Maki G., Zervos M. (2021). Health Care–Acquired Infections in Low- and Middle-Income Countries and the Role of Infection Prevention and Control. Infect. Dis. Clin. N. Am..

[280] Scott N.B., Pocock N.S. (2021). The Health Impacts of Hazardous Chsemical Exposures among Child Labourers in Low- and Middle-Income Countries. Int. J. Environ. Res. Public Health.

[281] Justo N., Espinoza M.A., Ratto B., Nicholson M., Rosselli D., Ovcinnikova O., Martí S.G., Ferraz M.B., Langsam M., Drummond M.F. (2019). Real-World Evidence in Healthcare Decision Making: Global Trends and Case Studies From Latin America. Value Health.

[282] Daniele M.A.S., Cleland J., Benova L., Ali M. (2017). Provider and lay perspectives on intra-uterine contraception: A global review. Reprod. Health.

[283] De Guzman R., Schiller J. (2025). Air pollution and its impact on cancer incidence, cancer care and cancer outcomes. BMJ Oncol..

[284] Shankar A., Saini D., Roy S. (2022). Air pollution and cancer. Ann. Oncol. Res. Ther..

[285] Onyije F.M., Hosseini B., Togawa K., Schüz J., Olsson A. (2021). Cancer Incidence and Mortality among Petroleum Industry Workers and Residents Living in Oil Producing Communities: A Systematic Review and Meta-Analysis. Int. J. Environ. Res. Public Health.

[286] Fagbohun T.R., Nji Q.N., Okechukwu V.O., Adelusi O.A., Nyathi L.A., Awong P., Njobeh P.B. (2025). Aflatoxin Exposure in Immunocompromised Patients: Current State and Future Perspectives. Toxins.

[287] Ekwomadu T., Mwanza M., Musekiwa A. (2022). Mycotoxin-Linked Mutations and Cancer Risk: A Global Health Issue. Int. J. Environ. Res. Public Health.

[288] Kehm R.D., Lloyd S.E., Burke K.R., Terry M.B. (2025). Advancing environmental epidemiologic methods to confront the cancer burden. Am. J. Epidemiol..

[289] Gomes D., Stavropoulou C. (2019). The impact generated by publicly and charity-funded research in the United Kingdom: A systematic literature review. Health Res. Policy Syst..

[290] Casini A., Pöthig A. (2024). Metals in Cancer Research: Beyond Platinum Metallodrugs. ACS Cent. Sci..

[291] Pal S., Firdous S.M. (2024). Unraveling the role of heavy metals xenobiotics in cancer: A critical review. Discov. Oncol..

[292] Ndagi U., Mhlongo N., Soliman M. (2017). Metal complexes in cancer therapy—An update from drug design perspective. Drug Des. Devel. Ther..

[293] Florea A.-M., Büsselberg D. (2011). Metals and Breast Cancer: Risk Factors or Healing Agents?. J. Toxicol..

[294] Firmani G., Chiavarini M., Dolcini J., Quarta S., D’Errico M.M., Barbadoro P. (2024). The Association Between Cadmium Exposure and Prostate Cancer: An Updated Systematic Review and Meta-Analysis. Int. J. Environ. Res. Public Health.

[295] Bede-Ojimadu O., Nnamah N., Onuegbu J., Grant-Weaver I., Barraza F., Orakwe J., Abiahu J., Orisakwe O.E., Nriagu J. (2023). Cadmium exposure and the risk of prostate cancer among Nigerian men: Effect modification by zinc status. J. Trace Elem. Med. Biol..

[296] Chatterjee M., Kortenkamp A. (2022). Cadmium exposures and deteriorations of cognitive abilities: Estimation of a reference dose for mixture risk assessments based on a systematic review and confidence rating. Environ. Health.

[297] Huang S., Kuang J., Zhou F., Jia Q., Lu Q., Feng C., Yang W., Fan G. (2019). The association between prenatal cadmium exposure and birth weight: A systematic review and meta-analysis of available evidence. Environ. Pollut..

[298] United States Environmental Protection Agency (1987). Guidelines for Delineation of Wellhead Protection Areas. J. Geosci. Environ. Prot..

[299] OEHHA (1987). Proposition 65 List of Chemicals, OEHHA. https://oehha.ca.gov/proposition-65/proposition-65-list.

[300] Laube B., Michaelsen S., Meischner V., Hartwig A., Epe B., Schwarz M. (2019). Classification or non-classification of substances with positive tumor findings in animal studies: Guidance by the German MAK commission. Regul. Toxicol. Pharmacol..

[301] Wogan G.N., Kensler T.W., Groopman J.D. (2012). Present and future directions of translational research on aflatoxin and hepatocellular carcinoma. A review. Food Addit. Contam. Part A.

[302] Kensler T.W., Roebuck B.D., Wogan G.N., Groopman J.D. (2011). Aflatoxin: A 50-Year Odyssey of Mechanistic and Translational Toxicology. Toxicol. Sci..

[303] Xu M., Xu Y., Huang Y., Shi J., Yin L., Tu J., Yin J., Zou C. (2025). Impact of environmental pollution on human health: Investigating the role of Polycyclic Aromatic Hydrocarbons in pediatric osteosarcoma. Ecotoxicol. Environ. Saf..

[304] Kotha S.V., Kuo G., Kammula S.V., Shi L., Zhang X., Liu P., Mao X. (2025). The Epidemiological and Toxicological Intersection of Air Pollution and Dementia. Rev. Environ. Contam. Toxicol..

[305] Parousis-Paraskevas O., Gkikoudi A., Al-Qaaod A., Vasilopoulos S.N., Manda G., Beinke C., Haghdoost S., Terzoudi G.I., Krasniqi F., Georgakilas A.G. (2025). Combined Radiations: Biological Effects of Mixed Exposures Across the Radiation Spectrum. Biomolecules.

[306] Zhu Z., Shen J., Ho P.C.-L., Hu Y., Ma Z., Wang L. (2025). Transforming cancer treatment: Integrating patient-derived organoids and CRISPR screening for precision medicine. Front. Pharmacol..

[307] Liu J., Gan T., Hu W., Li Y. (2024). Current status and perspectives in environmental oncology. Chronic Dis. Transl. Med..

[308] Jin K., Zhu F., Wu B., Li M., Wang X., Cheng X., Li M., Huang D., Xing C. (2024). Leukemia risk assessment of exposure to low-levels of benzene based on the linearized multistage model. Front. Public Health.

[309] Singh A.V., Bhardwaj P., Laux P., Pradeep P., Busse M., Luch A., Hirose A., Osgood C.J., Stacey M.W. (2024). AI and ML-based risk assessment of chemicals: Predicting carcinogenic risk from chemical-induced genomic instability. Front. Toxicol..

[310] Jiang M., Cai N., Hu J., Han L., Xu F., Zhu B., Wang B. (2025). Genomic and algorithm-based predictive risk assessment models for benzene exposure. Front. Public Health.

[311] Kang H., Liu X., Ge D., Zeng Y. (2025). Revolutionizing bladder cancer research: Harnessing 3D organoid technology to decode tumor heterogeneity and propel personalized therapeutics. Biochim. Biophys. Acta-Rev. Cancer.

[312] Jiang Y., Sun X., Song X., Li Z., Zhang P., Zhang W., Tang D. (2023). Patient-derived bladder cancer organoid model to predict sensitivity and feasibility of tailored precision therapy. Curr. Urol..

[313] Allenspach K., Zavros Y., Elbadawy M., Zdyrski C., Mochel J.P. (2023). Leveraging the predictive power of 3D organoids in dogs to develop new treatments for man and man’s best friend. BMC Biol..

[314] Wang B., Chen J.-Z., Luo X.-Q., Wan G.-H., Tang Y.-L., Wang Q.-P. (2022). The application of genome-wide CRISPR-Cas9 screens to dissect the molecular mechanisms of toxins. Comput. Struct. Biotechnol. J..

[315] Nnachi R.C., Sui N., Ke B., Luo Z., Bhalla N., He D., Yang Z. (2022). Biosensors for rapid detection of bacterial pathogens in water, food and environment. Environ. Int..

[316] Rainbow J., Sedlackova E., Jiang S., Maxted G., Moschou D., Richtera L., Estrela P. (2020). Integrated Electrochemical Biosensors for Detection of Waterborne Pathogens in Low-Resource Settings. Biosensors.

[317] Ramos-Sono D., Laureano R., Rueda D., Gilman R.H., La Rosa A., Ruiz J., León R., Sheen P., Zimic M. (2020). An electrochemical biosensor for the detection of Mycobacterium tuberculosis DNA from sputum and urine samples. PLoS ONE.

[318] Bernasconi S., Angelucci A., Rossi A., Aliverti A. (2025). A New Wearable System for Personal Air Pollution Exposure Estimation: Pilot Observational Study. JMIR MHealth UHealth.

[319] Dons E., Laeremans M., Orjuela J.P., Avila-Palencia I., Carrasco-Turigas G., Cole-Hunter T., Anaya-Boig E., Standaert A., De Boever P., Nawrot T. (2017). Wearable Sensors for Personal Monitoring and Estimation of Inhaled Traffic-Related Air Pollution: Evaluation of Methods. Environ. Sci. Technol..

[320] Andrews I., Dunn P., Hayler J., Hinkley B., Hughes D., Kaptein B., Lorenz K., Mathew S., Rammeloo T., Wang L. (2011). Green Chemistry Articles of Interest to the Pharmaceutical Industry. Org. Process. Res. Dev..

[321] Wang J., Cong Y., Tang B., Liu J., Pu K. (2025). Integrative analysis of multi-omics data and gut microbiota composition reveals prognostic subtypes and predicts immunotherapy response in colorectal cancer using machine learning. Sci. Rep..

[322] Qin Y., Wang Q., Lin Q., Liu F., Pan X., Wei C., Chen J., Huang T., Fang M., Yang W. (2025). Multi-omics analysis reveals associations between gut microbiota and host transcriptome in colon cancer patients. MSystems.

[323] Sanches P.H.G., de Melo N.C., Porcari A.M., de Carvalho L.M. (2024). Integrating Molecular Perspectives: Strategies for Comprehensive Multi-Omics Integrative Data Analysis and Machine Learning Applications in Transcriptomics, Proteomics, and Metabolomics. Biology.

[324] Mezynska M., Brzóska M.M. (2018). Environmental exposure to cadmium—A risk for health of the general population in industrialized countries and preventive strategies. Environ. Sci. Pollut. Res..

[325] Fanfani A., Papini S., Bortolotti E., Vagnoni G., Saieva C., Bonaccorsi G., Caini S. (2024). Cadmium in biological samples and site-specific cancer risk and mortality: A systematic review of original articles and meta-analyses. Cancer Epidemiol..

[326] Bartnicka J.J., Dyba T., Mezquita F.Y., Rasero F.R., Randi G., Jones A., Carvalho R. (2023). Lung cancer mortality and soil content of arsenic and cadmium: An ecological study in 26 EU countries. Eur. J. Public Health.

[327] Si W., Shen J., Zheng H., Fan W. (2019). The role and mechanisms of action of microRNAs in cancer drug resistance. Clin. Epigenet..

[328] Kensler T.W., Eaton D.L. (2024). 65 Years on—Aflatoxin Biomarkers Blossoming: Whither Next?. Toxins.

[329] McCullough A.K., Lloyd R.S. (2019). Mechanisms underlying aflatoxin-associated mutagenesis—Implications in carcinogenesis. DNA Repair.

[330] Rani R., Kela A., Dhaniya G., Arya K., Tripathi A.K., Ahirwar R. (2021). Circulating microRNAs as biomarkers of environmental exposure to polycyclic aromatic hydrocarbons: Potential and prospects. Environ. Sci. Pollut. Res..

[331] Amossé J., Souki R., El Hajjar M., Marques M., Genêt V., Février A., Le Gall M., SaintPierre B., Letourneur F., Le Ferrec E. (2024). Exploration of microRNAs from blood extracellular vesicles as biomarkers of exposure to polycyclic aromatic hydrocarbons. Ecotoxicol. Environ. Saf..

[332] Letelier P., Saldías R., Loren P., Riquelme I., Guzmán N. (2023). MicroRNAs as Potential Biomarkers of Environmental Exposure to Polycyclic Aromatic Hydrocarbons and Their Link with Inflammation and Lung Cancer. Int. J. Mol. Sci..

[333] Dasí-Navarro N., Lombardi S., Vila-Donat P., Llop S., Vioque J., Soler-Blasco R., Esplugues A., Manyes L., Lozano M. (2025). Metabolomic Profiling of Human Urine Related to Mycotoxin Exposure. Toxin.

[334] Paganelli A., Righi V., Tarentini E., Magnoni C. (2022). Current Knowledge in Skin Metabolomics: Updates from Literature Review. Int. J. Mol. Sci..

[335] Kim M., Lee S., Hur J., Shin D. (2025). Metabolomics and nutrient intake reveal metabolite–nutrient interactions in metabolic syndrome: Insights from the Korean Genome and Epidemiology Study. Nutr. J..

[336] Chen X., Zou G., Yang Z., Qi X., Song F., Peng L., Wang D., Zhou J., Ma J., He H. (2025). Serum metabolomic profiling uncovered metabolic shifts in individuals upon moderate-altitude exposure and identified the potentiality of beta-alanine to ameliorate hyperuricemia. Redox Biol..

[337] Chunduri V., Maddi S. (2022). Role of in vitro two-dimensional (2D) and three-dimensional (3D) cell culture systems for ADME-Tox screening in drug discovery and development: A comprehensive review. ADMET DMPK.

[338] Suarez-Martinez E., Suazo-Sanchez I., Celis-Romero M., Carnero A. (2022). 3D and organoid culture in research: Physiology, hereditary genetic diseases and cancer. Cell Biosci..

[339] Woo L.L., Egner P.A., Belanger C.L., Wattanawaraporn R., Trudel L.J., Croy R.G., Groopman J.D., Essigmann J.M., Wogan G.N. (2011). Aflatoxin B1-DNA Adduct Formation and Mutagenicity in Livers of Neonatal Male and Female B6C3F1 Mice. Toxicol. Sci..

[340] Hamid A.S., Tesfamariam I.G., Zhang Y., Zhang Z.G. (2013). Aflatoxin B1-induced hepatocellular carcinoma in developing countries: Geographical distribution, mechanism of action and prevention. Oncol. Lett..

[341] Garcia A.L.C., Kucab J.E., Al-Serori H., Beck R.S.S., Bellamri M., Turesky R.J., Groopman J.D., Francies H.E., Garnett M.J., Huch M. (2024). Tissue Organoid Cultures Metabolize Dietary Carcinogens Proficiently and Are Effective Models for DNA Adduct Formation. Chem. Res. Toxicol..

[342] Turkington R.E., Hukriede N.A., Ho J., Jayasundara N., Sanders A.P. (2025). Metal mechanisms of mitochondrial toxicity: Recent review of arsenic, cadmium, and lead-induced nephrotoxicity. Environ. Sci. Pollut. Res..

[343] Bridgeman L., Pamies D., Frangiamone M. (2025). Human organoids to assess environmental contaminants toxicity and mode of action: Towards New Approach Methodologies. J. Hazard. Mater..

[344] Qu F., Zheng W. (2024). Cadmium Exposure: Mechanisms and Pathways of Toxicity and Implications for Human Health. Toxics.

[345] Jeong S.H., Jung J., Park Y.-J., Lee S.J., Lee S.-J. (2025). The Influence of polycyclic aromatic hydrocarbons exposure on the gut microbiome composition and inflammatory responses. Ecotoxicol. Environ. Saf..

[346] Lv M., Chen S., Qin H., Wang Y., Liu Y., Liu R., Chen L., Qu G., Jiang G. (2025). Transformation of Bisphenols by Gut Microbiota: Insights into Species-Specific Pathways and Toxicity Implications. Environ. Sci. Technol..

[347] Aguayo F., Tapia J.C., Calaf G.M., Muñoz J.P., Osorio J.C., Guzmán-Venegas M., Moreno-León C., Levican J., Andrade-Madrigal C. (2025). The Role of Xenobiotics and Anelloviruses in Colorectal Cancer: Mechanisms and Perspectives. Int. J. Mol. Sci..

[348] Ansori A.N., Antonius Y., Susilo R.J., Hayaza S., Kharisma V.D., Parikesit A.A., Zainul R., Jakhmola V., Saklani T., Rebezov M. (2023). Application of CRISPR-Cas9 genome editing technology in various fields: A review. Narra J..

[349] Covarrubias S., Vollmers A.C., Capili A., Boettcher M., Shulkin A., Correa M.R., Halasz H., Robinson E.K., O’Briain L., Vollmers C. (2020). High-Throughput CRISPR Screening Identifies Genes Involved in Macrophage Viability and Inflammatory Pathways. Cell Rep..

[350] Zhang J., Hu S., Zhao C., Zhou Y., Zhang L., Liu H., Zhou P., Li S., Fu L., Zheng Z. (2022). Genome-Scale CRISPR Knockout Screening Identifies BACH1 as a Key Regulator of Aflatoxin B1-Induced Oxidative Damage. Antioxidants.

[351] Adam M.A.A., Kamal L.Z.M., Kanakal M., Babu D., Dahham S.S., Tabana Y., Lok B., Bermoy B.M., Yunus M.A., Than L.T.L. (2022). The Effect of Aflatoxin B1 on Tumor-Related Genes and Phenotypic Characters of MCF7 and MCF10A Cells. Int. J. Mol. Sci..

[352] Biederstädt A., Basar R., Park J.-M., Uprety N., Shrestha R., Silva F.R., Dede M., Watts J., Acharya S., Xiong D. (2025). Genome-wide CRISPR screens identify critical targets to enhance CAR-NK cell antitumor potency. Cancer Cell.

[353] Lalosevic M.S., Coric V., Pekmezovic T., Simic T., Markovic A.P., Ercegovac M.P. (2024). GSTM1 and GSTP1 Polymorphisms Affect Outcome in Colorectal Adenocarcinoma. Medicina.

[354] Wang Y., Yang Q., Zheng L. (2023). Association of oxidative stress, programmed cell death, GSTM1 gene polymorphisms, smoking and the risk of lung carcinogenesis: A two-step Mendelian randomization study. Front. Physiol..

[355] Hagger J.A., Depledge M.H., Oehlmann J., Jobling S., Galloway T.S. (2006). Is There a Causal Association between Genotoxicity and the Imposex Effect?. Environ. Health Perspect..

[356] Chappell G., Pogribny I.P., Guyton K.Z., Rusyn I. (2016). Epigenetic alterations induced by genotoxic occupational and environmental human chemical carcinogens: A systematic literature review. Mutat. Res. Mutat. Res..

[357] Lin Z., Chou W.-C. (2022). Machine Learning and Artificial Intelligence in Toxicological Sciences. Toxicol. Sci..

[358] Vosoughi P., Naghib S.M., Takdehghan G. (2025). Machine learning in cancer prognostic and diagnostic biomarkers: A promising approach for early cancer detection. Sens. Actuators Rep..

[359] Akkari I., Akkari H., Harbi R. (2025). Artificial intelligence to predict hepatocellular carcinoma risk in cirrhosis. World J. Gastrointest. Oncol..

[360] Gamlath C.J., Wu F. (2025). AI and Biotechnology to Combat Aflatoxins: Future Directions for Modern Technologies in Reducing Aflatoxin Risk. Toxins.

[361] Midya V., Alcala C.S., Rechtman E., Gregory J.K., Kannan K., Hertz-Picciotto I., Teitelbaum S.L., Gennings C., Rosa M.J., Valvi D. (2023). Machine Learning Assisted Discovery of Interactions between Pesticides, Phthalates, Phenols, and Trace Elements in Child Neurodevelopment. Environ. Sci. Technol..

[362] Limbu S., Glasgow E., Block T., Dakshanamurthy S. (2024). A Machine-Learning-Driven Pathophysiology-Based New Approach Method for the Dose-Dependent Assessment of Hazardous Chemical Mixtures and Experimental Validations. Toxics.

[363] Jiao Z., Hu P., Xu H., Wang Q. (2020). Machine Learning and Deep Learning in Chemical Health and Safety: A Systematic Review of Techniques and Applications. ACS Chem. Health Saf..

[364] Shi H., Kowalczewski A., Vu D., Liu X., Salekin A., Yang H., Ma Z. (2024). Organoid intelligence: Integration of organoid technology and artificial intelligence in the new era of in vitro models. Med. Nov. Technol. Devices.

[365] Wang Q., Guo F., Jin Y., Ma Y. (2022). Applications of human organoids in the personalized treatment for digestive diseases. Signal Transduct. Target. Ther..

[366] Bai L., Wu Y., Li G., Zhang W., Zhang H., Su J. (2024). AI-enabled organoids: Construction, analysis, and application. Bioact. Mater..

[367] Pricopoaia O., Cristache N., Lupașc A., Iancu D. (2025). The implications of digital transformation and environmental innovation for sustainability. J. Innov. Knowl..

[368] Lin X., Luo J., Liao M., Su Y., Lv M., Li Q., Xiao S., Xiang J. (2022). Wearable Sensor-Based Monitoring of Environmental Exposures and the Associated Health Effects: A Review. Biosensors.

[369] Srivastav A.K., Singh A., Singh S., Rivers B., Lillard J.W., Singh R. (2025). Revolutionizing Oncology Through AI: Addressing Cancer Disparities by Improving Screening, Treatment, and Survival Outcomes via Integration of Social Determinants of Health. Cancers.

[370] Cabral-Miranda W., Beloni C., Lora F., Afonso R., Araújo T., Fernandes F. (2025). Artificial intelligence platform to predict children’s hospital care for respiratory disease using clinical, pollution, and climatic factors. J. Glob. Health.

[371] Vargas-Santiago M., León-Velasco D.A., Maldonado-Sifuentes C.E., Chanona-Hernandez L. (2025). A State-of-the-Art Review of Artificial Intelligence (AI) Applications in Healthcare: Advances in Diabetes, Cancer, Epidemiology, and Mortality Prediction. Computers.

[372] Moreira A., Guedes J., da Silva M.V. (2025). New Methodologies and Techniques for Biomonitoring Pesticide Exposure in Agricultural Workers: A Systematic Review. Toxics.

[373] Anchidin-Norocel L., Bosancu A., Iatcu O.C., Lobiuc A., Covasa M. (2025). Real-Time Detection of Heavy Metals and Some Other Pollutants in Wastewater Using Chemical Sensors: A Strategy to Limit the Spread of Antibiotic-Resistant Bacteria. Chemosensors.

[374] Fagundes T.R., Coradi C., Vacario B.G.L., de Morais Valentim J.M.B., Panis C. (2025). Global Evidence on Monitoring Human Pesticide Exposure. J. Xenobiotics.

[375] Wang X., Bouzembrak Y., Lansink A.G.J.M.O., van der Fels-Klerx H.J. (2022). Designing a monitoring program for aflatoxin B1 in feed products using machine learning. Npj Sci. Food.

[376] Inglis A., Parnell A.C., Subramani N., Doohan F.M. (2024). Machine Learning Applied to the Detection of Mycotoxin in Food: A Systematic Review. Toxins.

[377] Liu J., Liu C., Liu Z., Zhou Y., Li X., Yang Y. (2025). Spatial analysis of air pollutant exposure and its association with metabolic diseases using machine learning. BMC Public Health.

[378] Kurul F., Doruk B., Topkaya S.N. (2025). Principles of green chemistry: Building a sustainable future. Discov. Chem..

[379] Lacourt C., Mukherjee K., Garthoff J., O’Sullivan A., Meunier L., Fattori V. (2024). Recent and emerging food packaging alternatives: Chemical safety risks, Current regulations, and analytical challenges. Compr. Rev. Food Sci. Food Saf..

[380] Pirzada T., de Farias B.V., Mathew R., Guenther R.H., Byrd M.V., Sit T.L., Pal L., Opperman C.H., Khan S.A. (2020). Recent advances in biodegradable matrices for active ingredient release in crop protection: Towards attaining sustainability in agriculture. Curr. Opin. Colloid Interface Sci..

[381] Mawcha K.T., Malinga L., Muir D., Ge J., Ndolo D. (2025). Recent Advances in Biopesticide Research and Development with a Focus on Microbials. F1000Research.

[382] Ediagbonya T.F., Areo I.O., Mupenzi C., Mind’je R., Kamuhanda J.K., Kabano S. (2025). Reduced pesticide dependency through crop management. Discov. Appl. Sci..

[383] Campanale C., Massarelli C., Savino I., Locaputo V., Uricchio V.F. (2020). A Detailed Review Study on Potential Effects of Microplastics and Additives of Concern on Human Health. Int. J. Environ. Res. Public Health.

[384] Sheriff S.S., Yusuf A.A., Akiyode O.O., Hallie E.F., Odoma S., Yambasu R.A., Thompson-Williams K., Asumana C., Gono S.Z., Kamara M.A. (2025). A comprehensive review on exposure to toxins and health risks from plastic waste: Challenges, mitigation measures, and policy interventions. Waste Manag. Bull..

[385] Khoshakhlagh A.H., Ghobakhloo S., Gruszecka-Kosowska A. (2024). Inhalational exposure to heavy metals: Carcinogenic and non-carcinogenic risk assessment. J. Hazard. Mater. Adv..

[386] Elnabi M.K.A., Elkaliny N.E., Elyazied M.M., Azab S.H., Elkhalifa S.A., Elmasry S., Mouhamed M.S., Shalamesh E.M., Alhorieny N.A., Elaty A.E.A. (2023). Toxicity of Heavy Metals and Recent Advances in Their Removal: A Review. Toxics.

[387] Slootweg J.C. (2024). Sustainable chemistry: Green, circular, and safe-by-design. One Earth.

[388] Charkiewicz A.E., Omeljaniuk W.J., Nowak K., Garley M., Nikliński J. (2023). Cadmium Toxicity and Health Effects—A Brief Summary. Molecules.

[389] Pan Z., Gong T., Liang P. (2024). Heavy Metal Exposure and Cardiovascular Disease. Circ. Res..

[390] Vaidya S.P., Gadre S., Kamisetti R.T., Patra M. (2022). Challenges and opportunities in the development of metal-based anticancer theranostic agents. Biosci. Rep..

[391] Khafaga D.S.R., El-Morsy M.T., Faried H., Diab A.H., Shehab S., Saleh A.M., Ali G.A.M. (2024). Metal–organic frameworks in drug delivery: Engineering versatile platforms for therapeutic applications. RSC Adv..

[392] Zong Z., Tian G., Wang J., Fan C., Yang F., Guo F. (2022). Recent Advances in Metal–Organic-Framework-Based Nanocarriers for Controllable Drug Delivery and Release. Pharmaceutics.

[393] Nerin C., Boobis A.R., Debarata K., Dubail S., Gude T., Kirchnawy C., Knaup B., Korzeniowski K.J., Lacourt C., Vitrac O. (2025). Review of potential areas for global harmonization of risk assessment protocols for Food Contact Materials (FCMs). Trends Food Sci. Technol..

[394] Baker J.L., Gordon-Dseagu V.L., Voortman T., Chan D., Herceg Z., Robinson S., Norat T., Croker H., Ong K., Kampman E. (2025). Lifecourse research in cancer: Context, challenges, and opportunities when exploring exposures in early life and cancer risk in adulthood. Health Open Res..

[395] Carlin D.J., Rider C.V. (2024). Combined Exposures and Mixtures Research: An Enduring NIEHS Priority. Environ. Health Perspect..

[396] Adegbola P.I., Adetutu A. (2024). Genetic and epigenetic modulations in toxicity: The two-sided roles of heavy metals and polycyclic aromatic hydrocarbons from the environment. Toxicol. Rep..

[397] Colacci A., Corsini E., Jacobs M.N. (2025). Addressing Immune Response Dysfunction in an Integrated Approach for Testing and Assessment for Non-Genotoxic Carcinogens in Humans: A Targeted Analysis. Int. J. Mol. Sci..

[398] Pan Y., Peter R.M., Chou P., Dave P.D., Xu J., Shanner A., Sarwar M.S., Kong A.-N. (2025). Cancer-specific Regulation of Metabolic and Epigenetic Pathways by Dietary Phytochemicals. Pharm. Res..

[399] Calafat A.M. (2016). Contemporary Issues in Exposure Assessment Using Biomonitoring. Curr. Epidemiol. Rep..

[400] Tang S., Li T., Fang J., Chen R., Cha Y., Wang Y., Zhu M., Zhang Y., Chen Y., Du Y. (2021). The exposome in practice: An exploratory panel study of biomarkers of air pollutant exposure in Chinese people aged 60–69 years (China BAPE Study). Environ. Int..

[401] Viegas S., Jeddi M.Z., Hopf N.B., Bessems J., Palmen N., Galea K.S., Jones K., Kujath P., Duca R.-C., Verhagen H. (2020). Biomonitoring as an Underused Exposure Assessment Tool in Occupational Safety and Health Context—Challenges and Way Forward. Int. J. Environ. Res. Public Health.

[402] Brown R.B., Mielke J.G. (2025). Carcinogenesis Associated with Toxin Nephropathy: Proposed Mediation by Phosphate Toxicity. Cells.

[403] Harrison D.J., Doe J.E. (2021). The modification of cancer risk by chemicals. Toxicol. Res..

[404] Nair A. (2019). Publication bias—Importance of studies with negative results!. Indian J. Anaesth..

[405] Frampton G., Whaley P., Bennett M., Bilotta G., Dorne J.-L.C.M., Eales J., James K., Kohl C., Land M., Livoreil B. (2022). Principles and framework for assessing the risk of bias for studies included in comparative quantitative environmental systematic reviews. Environ. Evid..

[406] Dong M., Wang L., Hu N., Rao Y., Wang Z., Zhang Y. (2025). Integration of multi-omics approaches in exploring intra-tumoral heterogeneity. Cancer Cell Int..

[407] Novak R., Robinson J.A., Frantzidis C., Sejdullahu I., Persico M.G., Kontić D., Sarigiannis D., Kocman D. (2023). Integrated assessment of personal monitor applications for evaluating exposure to urban stressors: A scoping review. Environ. Res..

[408] Joachim C., Vestris M., Marous M., Almont T., Ulric-Gervaise S., Dramé M., Contaret C., Smith-Ravin J., Escarmant P., Sylvestre E. (2020). Modeling the future of cancer registration and research: The Martinique Cancer Data Hub Platform. J. Glob. Health.

[409] Lee D.-H., Jacobs D.R. (2019). New approaches to cope with possible harms of low-dose environmental chemicals. J. Epidemiol. Community Health.

[410] Motsinger-Reif A.A., Reif D.M., Akhtari F.S., House J.S., Campbell C.R., Messier K.P., Fargo D.C., Bowen T.A., Nadadur S.S., Schmitt C.P. (2024). Gene-environment interactions within a precision environmental health framework. Cell Genom..

[411] Ho V., Pelland-St-Pierre L., Gravel S., Bouchard M.F., Verner M.-A., Labrèche F. (2022). Endocrine disruptors: Challenges and future directions in epidemiologic research. Environ. Res..

[412] Bardosh K.L., Ryan S.J., Ebi K., Welburn S., Singer B. (2017). Addressing vulnerability, building resilience: Community-based adaptation to vector-borne diseases in the context of global change. Infect. Dis. Poverty.

[413] Bodaghi A., Fattahi N., Ramazani A. (2023). Biomarkers: Promising and valuable tools towards diagnosis, prognosis and treatment of COVID-19 and other diseases. Heliyon.

[414] El-Tanani M., Rabbani S.A., El-Tanani Y., Matalka I.I., Khalil I.A. (2025). Bridging the gap: From petri dish to patient—Advancements in translational drug discovery. Heliyon.

[415] Ou F.-S., Michiels S., Shyr Y., Adjei A.A., Oberg A.L. (2021). Biomarker Discovery and Validation: Statistical Considerations. J. Thorac. Oncol..

[416] Klein W.M.P., O’Connell M.E., Bloch M.H., Czajkowski S.M., Green P.A., Han P.K.J., Moser R.P., Nebeling L.C., Vanderpool R.C. (2022). Behavioral Research in Cancer Prevention and Control: Emerging Challenges and Opportunities. JNCI J. Natl. Cancer Inst..

[417] Wang M., Yang H.-L., Liu X.-L., Mo B.-R., Kynoch K., Ramis M.-A. (2022). Evaluating behavioral economic interventions for promoting cancer screening uptake and adherence in targeted populations: A systematic review protocol. JBI Evid. Synth..

[418] Chen J., Guo H., Jiang H., Namusamba M., Wang C., Lan T., Wang T., Wang B. (2019). A BAP31 intrabody induces gastric cancer cell death by inhibiting p27 kip1 proteasome degradation. Int. J. Cancer.

[419] US EPA (2025). Integrated Risk Information System (IRIS) Chemical Assessments. https://www.epa.gov/iris.

[420] Battilani P., Palumbo R., Giorni P., Dall’Asta C., Dellafiora L., Gkrillas A., Toscano P., Crisci A., Brera C., De Santis B. (2020). Mycotoxin mixtures in food and feed: Holistic, innovative, flexible risk assessment modelling approach. EFSA Support. Public..

[421] Korchevskiy A.A., Wylie A.G. (2025). The IARC re-classification of talc carcinogenicity: A move in the wrong direction?. Crit. Rev. Toxicol..

[422] Goodson W.H., Lowe L., Carpenter D.O., Gilbertson M., Ali A.M., de Cerain Salsamendi L., Lasfar A., Carnero A., Azqueta A., Amedei A. (2015). Assessing the carcinogenic potential of low-dose exposures to chemical mixtures in the environment: The challenge ahead. Carcinogenesis.

