# Supplementary material for: Cancer and Environmental Xenobiotics: Mechanisms, Controversies, and Innovations
Source: J Xenobiot. 2025 Dec 19;16(1):2. doi: 10.3390/jox16010002 (PMC12821664; doi:10.3390/jox16010002)
Supplement: Supplementary file 1 [file jox-16-00002-s001.zip › jox-3938312-supplementary.pdf]

Supplementary Materials:

Table S1. IARC Classification of Key Environmental Carcinogens Included in This Review

Compound	CAS No.	Source / Relevance in Review	IARC Group	Key Notes
Aflatoxin B1 (AFB1)	1162-65-8	Major mycotoxin; gut–liver axis; carcinogenic metabolite	Group 1	Strong evidence in humans (liver cancer)
Ochratoxin A (OTA)	303-47-9	Foodborne mycotoxin; nephrotoxic; microbiome detoxication pathways	Group 2B	Sufficient animal evidence
Zearalenone (ZEA)	17924-92-4	Estrogenic mycotoxin; widely present in cereals	Group 3	Not classifiable
Deoxynivalenol (DON)	51481-10-8	Trichothecene mycotoxin; induces gut inflammation	Group 3	Limited mechanistic evidence
Fumonisin B1 (FB1)	116355-83-0	Mycotoxin linked to esophageal & liver cancer	Group 2B	Animal carcinogenicity (liver, kidney)
Ethanol (in alcoholic beverages)	64-17-5	Microbiome interaction; metabolite acetaldehyde	Group 1	Established human carcinogen
Acetaldehyde (from alcohol metabolism)	75-07-0	Bacterial & host metabolism; colon carcinogenesis	Group 1	Carcinogenic via DNA adducts
Benzo[a]pyrene (B[a]P)	50-32-8	Polycyclic aromatic hydrocarbon; diet; gut metabolism	Group 1	Strong animal & human evidence
N-nitrosodimethylamine (NDMA)	62-75-9	Nitrosamine; gut microbial involvement	Group 2A	Probable human carcinogen
N-nitrosodiethylamine (NDEA)	55-18-5	Food contaminants; endogenous formation	Group 2A	Strong mechanistic evidence
Heterocyclic amines (HCAs) – e.g., PhIP	105650-23-5	Formed during cooking; influenced by gut microbiome	Group 2B	Animal carcinogenicity
Benzene	71-43-2	Environmental contaminant; example for classification	Group 1	Included for comparison
1,3-Butadiene	106-99-0	Industrial pollutant; example of Group 1	Group 1	Included for context

Diethanolamine	111-42-2	Chemical exposure; relevant for classification example	Group 2B	Animal evidence
Cumene	98-82-8	Environmental PAH	Group 2B	Lung tumors in animals
Naphthalene	91-20-3	PAH; combustion & food smoke	Group 2B	Animal evidence

Sources: IARC. Agents Classified by the IARC Monographs and IARC Monographs on the Identification of Carcinogenic Hazards to Humans [418].

Key: International Agency for Research on Cancer (IARC) Monographs Classification System, classifies Group 1 – Carcinogenic to humans, Group 2A – Probably carcinogenic to humans, Group 2B – Possibly carcinogenic to humans, Group 3 – Not classifiable as to carcinogenicity in humans

Table S2. Toxicological Reference Values (Slope Factors) for Known and Probable Human Carcinogens: National and International Guidelines

Compound	CAS No.	IARC Group	Agency	Slope Factor	Units	Notes
Aflatoxin B1 (AFB1)	1162-65-8	Group 1	US EPA (IRIS)	1.5	$(mg/kg/day)^{-1}$	One of the highest known oral potency factors
			JECFA	0.3	$(mg/kg/day)^{-1}$	Based on margin-of-exposure evaluation
Ochratoxin A (OTA)	303-47-9	Group 2B	Not established	—	—	Cancer slope factor not officially set due to uncertainties
Zearalenone (ZEA)	17924-92-4	Group 3	Not established	—	—	Not classifiable; no slope factor
Deoxynivalenol (DON)	51481-10-8	Group 3	Not established	—	—	Threshold toxin; no cancer potency factor
Fumonisin B1 (FB1)	116355-83-0	Group 2B	US EPA (IRIS)	0.3	$(mg/kg/day)^{-1}$	Based on liver & kidney tumors in rodents
Ethanol (beverages)	64-17-5	Group 1	Not established	—	—	Cancer risk depends on ethanol metabolism → acetaldehyde

Acetaldehyde (from ethanol metabolism)	75-07-0	Group 1	US EPA (IRIS)	0.01	$(mg/kg/day)^{-1}$	Derived from inhalation data extrapolated to oral
Benzo[a]pyrene (B[a]P)	50-32-8	Group 1	US EPA (IRIS)	7.3	$(mg/kg/day)^{-1}$	Highly potent PAH carcinogen
			OEHHA (California)	1.1	$(mg/kg/day)^{-1}$	Based on updated state cancer potency values
N-nitrosodimethylamine (NDMA)	62-75-9	Group 2A	US EPA (IRIS)	51	$(mg/kg/day)^{-1}$	Very high potency factor; among highest
			OEHHA	10	$(mg/kg/day)^{-1}$	Based on drinking water assessment
N-nitrosodiethylamine (NDEA)	55-18-5	Group 2A	US EPA (IRIS)	150	$(mg/kg/day)^{-1}$	Even more potent; used for drinking water policy
Heterocyclic amines (PhIP)	105650-23-5	Group 2B	US EPA (HEAST)	0.42	$(mg/kg/day)^{-1}$	Cancer potency based on rodent bioassays
Benzene	71-43-2	Group 1	US EPA (IRIS)	0.055	$(mg/kg/day)^{-1}$	Based on leukemia risk
			OEHHA	0.1	$(mg/kg/day)^{-1}$	Updated CA value
1,3-Butadiene	106-99-0	Group 1	US EPA (IRIS)	3.0	$(mg/kg/day)^{-1}$	Derived from inhalation → oral extrapolation
Diethanolamine (DEA)	111-42-2	Group 2B	Not established	—	—	No cancer slope factor available
Cumene	98-82-8	Group 2B	US EPA (IRIS)	0.042	$(mg/kg/day)^{-1}$	Liver tumors in rats/mice
Naphthalene	91-20-3	Group 2B	Not established	—	—	No slope factor; mainly noncancer endpoints

Sources: United States Environmental Protection Agency Integrated Risk Information System (US EPA IRIS), California Office of Environmental Health Hazard Assessment (OEHHA), Health Canada (HC), European Food Safety Authority (EFSA), and the Joint FAO/WHO Expert Committee on Foocd Addictives (JECFA) [419,299,420,421,422]
